# Coronary artery disease and cardiac pathology in cerebral small vessel disease and post‐stroke dementia: Implications for Alzheimer's disease

**DOI:** 10.1002/alz.71979

**Published:** 2026-11-06

**Authors:** Yoshiki Hase, Tuomo Polvikoski, Dan Jobson, Klarizze Premarion, Ethan Tutter, Erika Kitajima, Pazhanichamy Kalailingam, Xavier Gallart‐Palau, Siu Kwan Sze, Louise Allan, Raj N. Kalaria

**Affiliations:** ^1^ Neurovascular Research Group, Translational and Clinical Research Institute Campus for Ageing & Vitality Newcastle University Newcastle Upon Tyne UK; ^2^ Center for Genomic Medicine Massachusetts General Hospital and Harvard Medical School Boston Massachusetts USA; ^3^ Biomedical Research Institute of Lleida Dr. Pifarré Foundation (IRBLLEIDA) ‐ +Pec Proteomics Research Group (+PPRG) ‐ Neuroscience Area, University Hospital Arnau de Vilanova (HUAV) Lleida Spain; ^4^ Faculty of Applied Health Sciences Brock University, St. Catharines Ontario Canada; ^5^ University of Exeter Medical School Exeter UK

**Keywords:** atherosclerosis, cardiac pathology, coronary artery disease, post‐stroke dementia, small vessel disease, vascular dementia

## Abstract

**BACKGROUND:**

The relationships between coronary or cardiac pathology, cerebral small vessel disease (SVD), Alzheimer's disease pathology, and vascular cognitive impairment and dementia (VCID) remain unclear.

**METHODS:**

We performed a clinicopathological study in 75 stroke patients enrolled in the prospective Cognitive Function After Stroke (CogFAST) cohort. Comprehensive histopathological examination was performed to characterize the spectrum of pathologies in samples of the main coronary arteries (CorA), and cardiac and brain tissues from stroke survivors diagnosed with post‐stroke dementia (PSD; mean age, standard deviation, range, % sex; 88 ± 8 years, 65–99, 55% female) or without dementia (PSND; 86 ± 4 years, 79–94, 45% female).

**RESULTS:**

All subjects exhibited occult or obstructive CorA disease with > 50% clinically defined stenosis in at least one of the main coronary arteries. Linear correlation analysis showed that CorA stenosis was positively related to internal carotid artery stenosis (*P* < 0.03). CorA stenosis was not associated with pathological measures of cerebral SVD per se or neurodegenerative disorders. Cardiac pathology was highly prevalent, with ≈ 80% of the subjects having at least one feature of cardiac pathology including fibrosis, disarray, and myocyte damage and 62% exhibiting myocardial infarcts. CorA stenosis was associated with higher cardiac pathology score, which was also related to cardiac sympathetic denervation and α‐synuclein accumulation in the stellate ganglia. We noted low heart weights (*P* < 0.001), greater myocardial infarcts (*P* < 0.001), and greater general cardiac atrophy (*P* = 0.024) in the PSD compared to the PSND group.

**CONCLUSIONS:**

Our findings suggest there is a relationship between CorA disease and carotid artery disease and that cardiac pathology in elderly stroke survivors may be a factor in the development of delayed dementia after the stroke injury and VCID.

## Highlights

- Correlation analysis showed that occult or obstructive coronary artery disease with > 50% clinically defined occlusion was positively related to internal carotid artery stenosis.
- The burden of cerebral infarcts was highest in patients with moderate stenosis in both the coronary arteries and carotid arteries but coronary artery stenosis was not associated with pathological measures of neurodegenerative pathology.
- Coronary artery stenosis was associated with higher cardiac pathology scores, which were also related to cardiac sympathetic denervation and α‐synuclein accumulation in the stellate ganglia.
- Low heart weights, greater myocardial infarcts, and general cardiac atrophy were associated with dementia in post‐stroke survivors compared to stroke patients who did not develop dementia.

## 1 INTRODUCTION

Coronary artery (CorA) disease is a major contributor to morbidity and mortality worldwide. Coronary and cardiac pathologies have the potential to significantly influence perfusion in the end organs, including the brain. CorA disease is also part and parcel of strokes because both conditions involve atherosclerosis and often occur together.^1^ A recent meta‐analysis^2^ suggested that an individual with any type of cardiovascular disease including CorA disease, atrial fibrillation (AF), and myocardial infarction (MI) had greater risk of developing Alzheimer's disease (AD). Thus, understanding this link between the heart and brain is important for diagnosis, prevention, and treatment of vascular disease as well as all‐cause dementia.

CorA disease and stroke share many major risk factors including hypertension, type 2 diabetes mellitus (T2DM), hyperlipidemia, smoking, high body mass index, and physical inactivity. These factors largely damage the arterial system to accelerate atheromatous plaque formation and promote stenosis. It is widely accepted that MI and arrhythmias or AF increase risk of emboli or microemboli, which may travel through carotid and vertebral arteries to cause small ischemic lesions within the spectrum of small vessel disease (SVD),^3^ although this view can be challenged.^4^

Previous studies suggest that 20% to 25% of patients with stroke have significant CorA disease. In a recent Norwegian study,^5^ the overall prevalence of CorA disease was 25% in 15‐ to 60‐year‐old stroke survivors but in those with obstructive CorA disease, defined as ≥ 50% stenosis in any segment of the major epicardial arteries, it was 15.6%. This report indicated that a remarkable quarter of young and middle‐aged ischemic stroke survivors had CorA disease and that CorA disease was an independent predictor of recurrent stroke and mortality.^5^ In another study, 18% of ischemic stroke patients had significant CorA disease.^6^ While T2DM was an independent factor of CorA disease there was no association between large artery disease and CorA disease, while another study reported that subclinical CorA disease defined by ≥ 50% stenotic disease with calcium deposits in coronary arteries was present in 21% of patients who had suffered a suspected embolic stroke.^7^ In a retrospective study when CorA disease was present, ischemic stroke was apparent in 32% of older subjects. Conversely, if ischemic stroke was present then CorA disease was also prevalent in 56% of subjects in age range 60 to 102 years.^8^ In this study, peripheral arterial disease was also reported to co‐occur with CorA disease (68%) and ischemic strokes (42%).

*Post mortem* studies have revealed even more staggering associations between CorA stenosis and stroke. An autopsy study^9^ reported that the prevalence of ≥ 50% coronary stenoses was 38% in 341 cases of fatal stroke whereas the frequency of coronary plaques was 72%. In comparison, CorA stenosis and plaques occurred in 27% and 10%, respectively, in 462 subjects with other neurological disorders. This study also reported that 41% of the cases had pathological evidence (macro and microscopic) of MI indicating that many coronary events are clinically silent and largely detected *post mortem*. The rate of presymptomatic or subclinical CorA disease in stroke patients who have never had cardiac symptoms is considered common,^7^, ^10^, ^11^ with further suggestions that CorA disease is also an indicator of systemic vascular disease not only involving carotid and intracranial arteries.^8^ However, the data are sparse on whether CorA disease is a marker of similar pathological changes within brain blood vessels. Emerging evidence suggests that damaged proteins in the blood vessel wall, such as isoaspartate–glycine–arginine (isoDGR) formation,^12^ may promote chronic inflammation and structural changes in arteries. These changes could provide a common link among coronary and carotid disease with brain blood vessels.

**RESEARCH IN CONTEXT**

1. **Systematic review**: The authors reviewed the relevant literature from PubMed and Scopus online databases. While previous studies have shown a relationship between carotid arteries and cerebral lesions in dementia there is limited knowledge on assessment of coronary arteries, carotid arteries, and cerebral lesions within the same patients and aging‐associated dementias.
2. **Interpretation**: Our report suggests there is a relationship between coronary artery disease and carotid artery disease, and that cardiac pathology in elderly stroke survivors is an important factor in delayed dementia after the stroke injury. These findings have implications for patients with Alzheimer's disease and for vascular cognitive impairment and dementia (VCID) who often exhibit moderate coronary and carotid artery stenosis. We suggest that systemic vascular pathology, particularly in the heart, contributes to cerebral hypoperfusion during aging.
3. **Future directions**: Our findings provide clinicopathological evidence to suggest that cardiovascular pathology is a compelling contributor to the development of dementia. Careful investigation of the pathophysiology of the cardiovascular system should provide more clues to readily reduce the burden of VCID and other dementias for which therapeutic approaches to tackle neurodegenerative pathologies have been challenging.

Here, we assessed the spectrum of CorA disease in relation to cardiac and cerebral SVD pathologies in prospectively collected cardiovascular and cerebral tissues from the Cognitive Function After Stroke (CogFAST) cohort. There are limited studies on the relationship or interaction between CorA disease and intracranial brain vascular or parenchymal pathology. Our general hypothesis was that cardiac pathologies impact the burden of cerebral SVD and dementias including those with AD type of pathology.

## 2 MATERIALS AND METHODS

### 2.1 Study design and subjects

Demographic, clinical and pathological findings in the 75 subjects of the study are given in Table 1. Study subjects were participants of the CogFAST study.^13^ They had a clinical diagnosis of post‐stroke no dementia (PSND) and post‐stroke with dementia (PSD). The entire PSD group was also categorized as vascular cognitive impairment and dementia (VCID).^14^ There are no cases in the PSND group that met the vascular cognitive impairment mild criteria. Mini‐Mental State Examination (MMSE; < 25) and Cambridge Cognitive Examination (CAMCOG; < 80) scores were obtained to define cognitive status along with Diagnostic and Statistical Manual of Mental Disorders 3rd Edition Revised to reach consensus for dementia.^13^ We previously ascertained that ≈ 78% of the PSD subjects^13^ were diagnosed as vascular dementia (VaD),^15^, ^16^ whereas the rest were mixed dementia (mixed) with AD and VaD features. However, all had strong clinical as well as pathological evidence of cerebral SVD including confluent white matter (WM) hyperintensities.^17^

**TABLE 1:** Severity of coronary artery disease in relation to clinical measures, cognition and brain pathology in the CogFAST cohort.

Variable	Total^a^	Mild	Moderate	Severe	Significance (***P*** value)^*^
No. of subjects	75 (100%)	23 (31%)	40 (53%)	12 (16%)	–
Age (years), mean ± SEM (range)	87.1 ± 0.7 (65–99)	84.9 ± 1.6 (65–96)	88.3 ± 0.9 (75–99)	87.5 ± 1.6 (79–97)	0.202
Sex (male)	48%	48%	44%	62%	0.533
Brain weight (g)	1232 ± 16	1189 ± 31	1236 ± 20	1278 ± 38	0.170
**% Clinical stenosis severity**		≤50	50‐69	≥70	–
% Area of coronary artery stenosis, mean ± SEM^c^ (range)	75.5 ± 1.1 (59–100)	64.8 ± 0.4 (59–68)	77.0 ± 0.8 (70–87)	91.3 ± 1.4 (85–100)	<0.001
**Clinical features**					
Dementia (PSD)^b^	55%	52%	61%	46%	0.622
Last MMSE	21.3 ± 0.9	22 ± 1	21 ± 1	22 ± 2	0.232
Last CAMCOG	71.9 ± 3	76 ± 4	69 ± 4	73 ± 6	0.464
Hypertension	57%	76%	53%	39%	0.232
Type 2 diabetes	7%	6%	7%	8%	0.981
Hypercholesterolemia	13%	6%	10%	31%	0.058
Peripheral vascular disease‖	8%	6%	6%	0.2%	0.368
Smoking history‖‖	64%	88%	50%	75%	0.284
Total no. of VRF (0–3)	1.62 ± 0.11	1.55 ± 0.15	1.58 ± 0.15	1.85 ± 0.34	0.617
No. of VRFs (≥ 1)	87%	94%	87%	77%	>0.05
No. of VRFs (≥ 2)	61%	59%	60%	69%	>0.05
No. of VRFs (≥ 3)	27%	18%	27%	39%	>0.05
**Carotid artery disease**					
% Carotid artery stenosis, mean ± SEM^d^(range)	63.8 ± 1.7 (32–96)	57.5 ± 4.3 (32–96)	65.3 ± 1.8 (44–87)	71.1 ± 3.0 (52–87)	<0.025
**Brain pathology markers**					
Braak stage, mean (range)	2.82 (0–6)	2.7 (0–6)	2.9 (0–6)	2.9 (1–5)	>0.05
Thal score, mean (range)	1.7 (0–3)	1.6 (0–3)	1.7 (0–3)	1.7 (0–3)	>0.05
CERAD score, mean (range)	1.14 (0–3)	0.80 (0–3)	1.2 (0–3)	1.4 (0–3)	>0.05
α‐synuclein, mean (range)	0.8 (0–14)	0.0 (0)	0.7 (0–6)	1.7 (0–14)	>0.05
CAA score, mean (range)	1.1 (0–3)	1.0 (0–3)	1.1 (0–3)	1.1 (0–3)	>0.05
SVD pathology score‖ (range)	13.2 ± 0.3 (5–17)	13.1 ± 0.6 (5–17)	13.0 ± 0.3 (8–17)	13.2 ± 0.3 (10–16)	0.969
Total cerebral infarcts	4.2 ± 0.3	4.1 ± 0.6	4.2 ± 0.5	4.7 ± 0.7	0.816
Cortical infarcts, mean (range)	2.0 (0–7)	1.9 (0–4)	2.1 (0–7)	2.0 (0–6)	0.925
Subcortical infarcts, mean (range)	2.4 (0–9)	2.6 (0–9)	2.2 (0–6)	2.7 (0–5)	0.604
Intracranial artery score^d^	4.1 ± 0.2	3.6 ± 0.5	4.2 ± 0.4	4.5 ± 0.7	0.499
WM scores	2.8 (1–3)	2.8 (1–3)	2.8 (1–3)	2.9 (1–3)	0.852

*Note*: Values represent mean ±standard error of the mean (SEM) of the scale variable, % feature within the group or mean scores (range) for the number of subjects shown.

Abbreviations: ANOVA, analysis of variance; Braak, Braak neurofibrillary tangle stage; CAA, cerebral amyloid angiopathy; CAMCOG, Cambridge Cognitive Examination; CERAD, Consortium to Establish a Registry for Alzheimer's Disease; CogFAST, Cognitive Function After Stroke; CorA, coronary artery; MMSE, Mini‐Mental State Examination; No., number; PSD, post‐stroke dementia; PSND, post‐stroke non‐dementia; SVD, small vessel disease; VRF, vascular risk factor; WM, white matter.

^a^ CorA stenosis was determined by taking the average of stenosis in the left descending, the right and left main coronary arteries (see Methods and Figure 1A).

^b^ Post‐stroke dementia (PSD) was defined by Diagnostic and Statistical Manual of Mental Disorders IV, IVR, or V criteria. PSD was not associated with severity of CorA stenosis. ‖Peripheral artery disease comprises either present or resolved pathologies; ‖‖Smoking history involves either ex‐smokers or current smokers. VRF analysis was limited to three factors including hypertension, diabetes and hypercholesterolemia.

^c^ ANOVA showed differences between mild, moderate, and severely stenosed subjects (*F* value 153.1, df 2, *P *< 0.001).

^d^ ANOVA showed differences between mild, moderate, and severely stenosed subjects (*F* value 4.0, df 2, *P *= 0.025).

To study and validate degrees of cardiac innervation, we determined α‐synuclien (αSYN) immunoreactivity in the stellate ganglion and tyrosine hydroxylase (TOH) as a marker of sympathetic reactivity in cardiac tissues in an additional 15 subjects. These had the following pathological diagnoses (mean age years ± standard error of the mean [SEM]; range; % male): 7 with AD (86 ± 3; 73–94; 43%), 6 with dementia with Lewy bodies or Parkinson's disease dementia (87 ± 3; 76–94; 33%), and 2 with mixed vascular and AD (90 ± 4; 86–93; 50%; Table 1). These were selected and added on the basis they exhibited mild to severe stellate ganglion pathology.^18^

Ethical approval and permissions for this study using donated human brains was granted by the Newcastle and North Tyneside 1 Research Ethics Committee and facilitated by the Newcastle Brain Tissue Resource (NBTR). Permission for use of cardiovascular tissues and brains for *post mortem* research was also granted by consent from the participants themselves, next of kin, or family member. All tissues were retained in and obtained from the NBTR.

### 2.2 Vascular risk factors and cardiac pathophysiology features

Patients enrolled in the CogFAST study had their medical histories reviewed from hospital records at baseline.^13^ Vascular risk factors (VRFs) included hypertension (a documented history of blood pressure > 140/90 mm Hg or treatment of hypertension), AF, ischemic heart disease (IHD), peripheral vascular disease, hypercholesterolemia, diabetes (documented or treated), and history of smoking prior to stroke (Table 1). Numbers of VRFs were calculated for each of the CorA stenosis groups and expressed as percent. The presence or absence of cardiovascular traits including infarction, IHD, heart failure, angina pectoris, and AF was recorded as a binary value and the percentage calculated for each degree of CorA stenosis.

### 2.3 *Post mortem* coronary arteries, carotid arteries, and brain tissues

Samples of the left descending (LD), right (RA), and left (LA) CorA were taken at 5 mm below the level of any bifurcations. For quantitative analysis, 10 µm thick serial sections cut from paraffin embedded transverse CorA blocks were stained with hematoxylin and eosin (H&E). Although carotid artery stenosis assessed by maximal diameters was categorized into mild, moderate, and severe based on previous modifications of the ultrasound and angiographic methods,^19^ for pathological quantification we chose to categorize CorA stenosis severity by % area and defined mild as < 70%, moderate as 71% to 89%, and severe as > 90% stenosis (Table 1; Figure 1).

**FIGURE 1:**
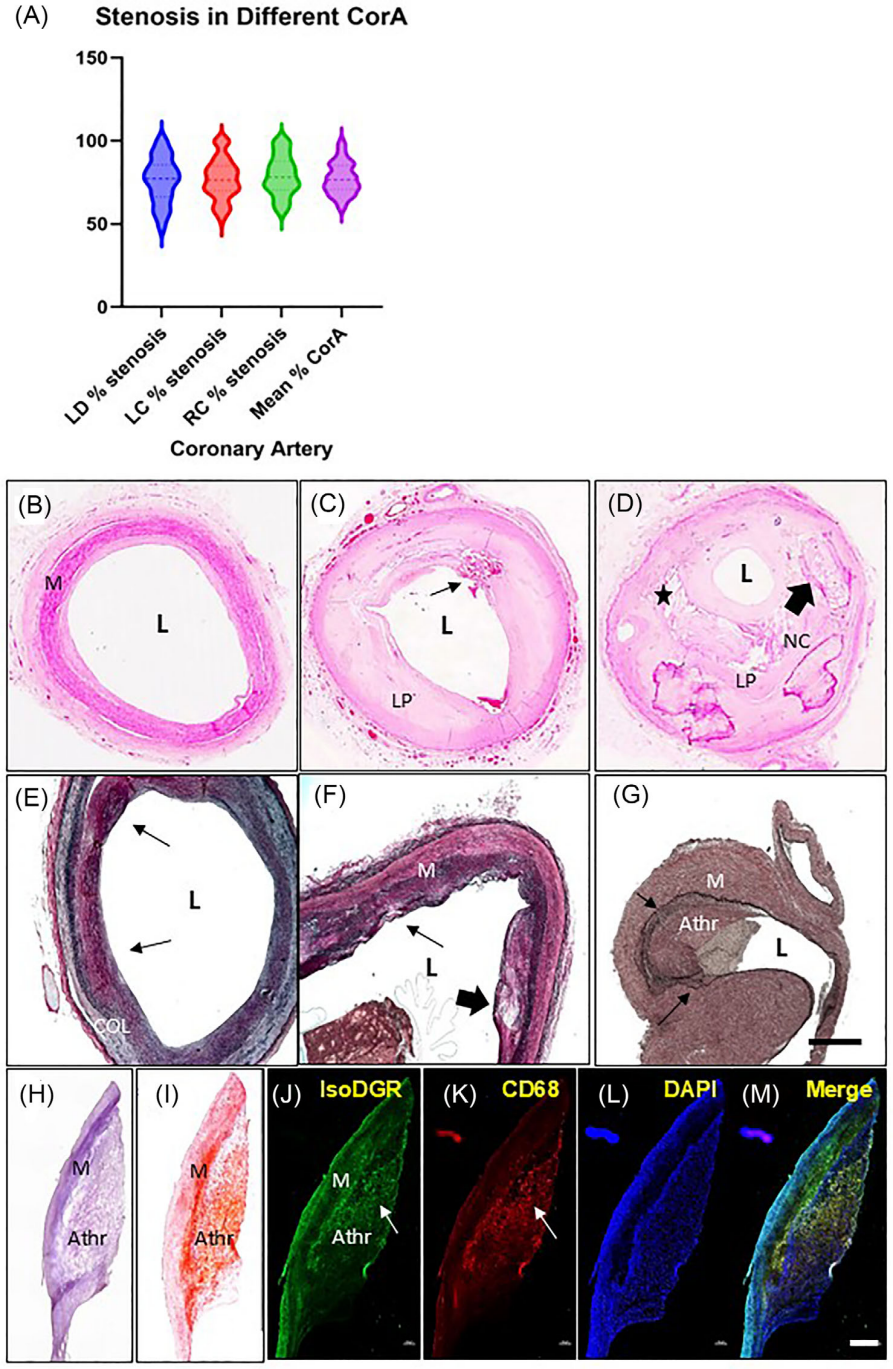
Coronary artery pathology in the CogFAST cohort. A, Violin plots showing the distribution of % stenosis in LD, LC, and RC arteries. The mean (± SEM) % stenosis of LD, LC, and RC arteries were 76 ± 2, 77 ± 2, 79 ± 2 (ANOVA *F* 0.672, df 2, *P *= 0.512). There were also no differences in mean % stenosis between right and the two left arteries (*P *> 0.05). The mean % stenosis incorporating all three arteries was calculated to be 77 ± 1.7. B–G, Coronary artery stenosis in post‐stroke patients. Representative images from sections stained with H&E show mild (B), moderate (C), and severe (D) degrees of stenosis and changes in walls of the main right coronary artery. Intimal thickening was most common in the mild cases whereas fibrocalcific lesions were most common (> 80%) in the moderate and severe stenosed arteries; (B) shows some intima thickening and fibrous media (M); in (C), thin arrow shows ruptured cap; (D) demonstrates complicated lesions with a necrotic core (NC), lipid pool (LP), calcified plaques (thick arrowhead), and cholesterol clefts (star). E–M, Representative sections of the left descending artery (E), right main coronary artery (F), and left main coronary artery (G–I) stained with EvG, H&E, and Oil Red. As above, (E–G) show arterial wall changes in the intima with thickening and disintegration (thin arrows in [E] and [F]), development of lipid cleft (thick arrow in [F]) and atheroma (Athr in [G–J]). G, In this artery, the junction of intima and media is clearly demarcated by the wrinkled elastic lamina (short arrow) and there is also evidence of reduplication. H–M, Representative tinctorial (EvG and Oil Red) and immunofluorescence images of atheromatous lesions in longitudinal sections of the left coronary artery. J–L, Sections stained with antibodies to isoDGR, CD68, and DAPI. M, Merged image illustrating degenerative protein modifications (isoDGR) co‐localized with CD68 immunostained macrophages (arrows) depicting cellular inflammation within the coronary artery. Scale bars represent 1 mm (B–G) and (H–M). ANOVA, analysis of variance; CogFAST, Cognitive Function After Stroke; COL, collagen; CorA, coronary artery; DAPI, 4′,6‐diamidino‐2‐phenylindole; df, degrees of freedom; EvG, Elastica Van Gieson; H&E, hematoxylin and eosin; isoDGR, isoaspartate–glycine–arginine; LC, left coronary; LD, left descending; RC, right coronary; SEM, standard error of the mean.

Samples of cardiac muscle from the atrioventricular node and left ventricle were taken at *post mortem* in most cases being reported in this study. Stellate ganglia were identified by their oval or “star” shape located anterior to the neck of the first rib and to the carotid artery. 10 µm thick cardiac tissue or stellate ganglion sections were stained for various tinctorial stains as specified to reveal cardiac and stellate ganglion pathology. In addition, samples of the cardiac muscle nerves with the (≈ 1 cm) ganglion were immunostained for TOH (TH‐16 clone, lot number 036M4828, Sigma; dilution 1:200) and αSYN (KM51 clone, Leica Biosystems; dilution 1:200).^18^

Brains were sampled bilaterally and assessed in accordance with the Newcastle brain dissection protocol. Standardized protocols were used for microscopic and macroscopic pathology assessment.^20^, ^21^ Briefly, macroscopic infarcts were recorded by visual inspection during dissection, and subsequently confirmed by microscopy. The size and total number of infarcts (vascular lesions) in both hemispheres in the cortex, basal ganglia, thalamus, WM, brainstem, and cerebellum were recorded as follows: < 5 mm, 5 to 15 mm, 16 to 30 mm, 31 to 50 mm, and > 51 mm. H&E stain was used for neuropathological assessment and inc vascular pathology scores to confirm SVD pathology. WM scores (0–3) were determined as described previously based on the degree of demyelination and vascular pathology.^17^, ^22^

### 2.4 CorA disease and measurement of CorA stenosis

Histopathological evaluation of CorA samples was performed using standard stains including H&E, Elastica Van Gieson and Masson's Trichrome.^23^, ^24^ Further cellular characterization of the CorA vessel walls and atheromatous material was undertaken by immunostaining for α‐smooth muscle actin (α‐SMA), CD68‐positive macrophages, and isoDGR‐associated degenerative protein modifications (1:200–1:1000 antibody dilutions).^25^, ^26^ Carotid arteries including the circle of Willis, basilar, and intracranial cerebral arteries were assessed and scored for pathological changes as described previously.^27^ CorA stenosis was measured as % area by scanning three to four 10 μ m thick H&E stained sections with the highest degree of stenosis using a photo scanner (EPSON Perfection V700, Seiko Epson Corporation).^28^ Total area of the external margin of the adventitia (S1) and luminal area at the interior margin of the intima (S2) were measured using ImageJ software (National Institutes of Health).

Stenosis in samples of the LD, RA, and LA coronary arteries were calculated essentially as described previously for internal carotid arteries.^27^ Briefly, % area stenosis was calculated using the following formula: ([*E – D*]/*E*) x 100 [%]), where *D* is the minimal area of the free lumen and *E* is the cross‐sectional area of the original vessel. As demonstrated previously,^27^ for example, 70% area stenosis corresponded to ≈ 50% luminal diameter measures per both the European Carotid Surgery Trial (ECST) and North American Symptomatic Carotid Endarterectomy Trial (NASCET). In addition, the Society of Cardiovascular Computed Tomography guidelines^19^ indicate that obstructive CorA disease is defined as equal to 50% stenosis in any segment of the major epicardial arteries, with high‐risk or occult CorA disease (prognostic lesions) as obstructive lesions in all three major arterial territories. Data analyses were performed by three investigators (Y.H., K.P., E.K.) blinded to case identities.

### 2.5 Brain pathology: neurodegenerative and vascular assessment

We used standard silver impregnation and tau immunohistochemistry (AT8 for phosphorylated tau at 1:1000 dilution) to assess neuritic plaques and neurofibrillary tangles for the Consortium to Establish a Registry for Alzheimer's Disease (CERAD) plaque score, Braak neurofibrillary tangle burden, and Thal score for amyloid beta (Aβ) staging.^29^ α‐SYN was the pathological alteration in Parkinson's disease dementia and dementia with Lewy bodies.^22^ Severity of cerebral amyloid angiopathy (CAA) was assessed using a 4‐point scale.^30^ SVD pathology scores (Table 1) were determined out of a total of 20 according to Deramecourt et al.^17^ In brief, SVD severity scores were rated in the temporal and frontal lobes (6 points each), hippocampus and basal ganglia including thalamus (4 points each) for degrees of arteriolosclerosis, CAA, perivascular hemosiderin leakage, perivascular spaces dilatation in the deep and juxtacortical WM, myelin loss, and cortical micro (< 0.5 cm) or large infarcts (> 0.5 cm), classified as either lacunar (cavitating infarcts measuring up to 15 mm) or territorial. Each lesion was graded according to severity using a semi‐quantitative scale ranging from 0 to 3 (0 = normal, 1 = mild changes, 2 = moderate lesions, and 3 = severe modification). The degrees of change were based upon the worst lesion in the section. Total cerebral, cortical, and subcortical infarcts, as well as intracranial artery and WM scores, were essentially determined as described previously.^27^

### 2.6 Cardiac pathology assessment

To ascertain cardiac pathology scores (Table 2), sections from the left ventricle were examined to determine the degrees of necrosis, fibrosis, and disarray. In several cases, we also examined sections from the atrioventricular and sinoventricular nodes. The total cardiac pathology score was determined by calculating the % area fibrosis, % area necrosis, and the presence of disarray as a binary number 0 (no) or 1 (yes). Myocardial sections which displayed the worst pathology were selected for each case for analysis. Percentage area of necrosis and fibrosis were classified into mild (< 20%), moderate (20%–50%) and severe (> 50%) based on a modified method used by Milano et al.^31^ The relative volumes of necrosis and fibrosis in the tissue sections were determined by point counting using a 10 × 10 grid at a total magnification of 100x. As previously performed,^31^ we did not differentiate between perivascular and intraparenchymal fibrosis, which reflects the total degree of fibrosis for each sample.

Because microscopic examination of the heart was limited mostly to the left ventricle, we verified the presence or absence of all infarcts irrespective of size also as a binary value 0 (no) or 1 (yes). The *Z* scores for each case were calculated from the values determined for the three markers of cardiac pathology: heart weight, cardiac pathology scores, and CorA stenosis. Scale data were first converted into a *Z* score for each individual marker and then a composite *Z* score was computed with each marker influencing the final *Z* score equally by 33.3%.

**TABLE 2:** Clinical and pathological indices of cardiac pathology in relation to coronary artery stenosis in CogFAST.

Variable (% area stenosis category)	Total	Mild	Moderate	Severe	Significance (*P* value)
Number of subjects	75 (100%)	23 (31%)	39 (52%)	13 (17%)	–
Age (years), mean ± SEM (range)	87.1 ± 0.7 (65–99)	84.9 ± 1.6 (65–96)	88.3 ± 0.9 (75–99)	87.5 ± 1.6 (79–97)	0.202
Sex (male %)	48%	48%	44%	62%	0.533
**Cardiac clinical features**					
Myocardial infarction	12%	0%	20%	8%	0.211
Ischemic heart disease	47%	29%	57%	46%	0.300
Cardiac failure	20%	12%	23%	23%	0.418
Angina pectoris	45%	29%	53%	46%	0.310
Atrial fibrillation	18%	24%	13%	23%	0.902
Cardiac traits (= 1)	59%	47%	63%	67%	>0.05
Cardiac traits (= 2)	53%	35%	57%	67%	>0.05
Cardiac traits (≥ 3)	22%	6%	33%	17%	>0.05
**Cardiac pathology**					
Heart weight (g) (range)	392 ± 14 (217–689)	373 ± 17 (217–689)	379 ± 17 (249–610)	449 ± 29 (296–653)	0.134
Total % cardiac pathology, mean ± SEM (range)	80 ± 2.2 (30–98)	78.9 ± 3.7 (60–98)	80.0 ± 3.4 (30–98)	87.2 ± 2.1 (75–95)	0.396
Myocardial infarcts (% lesions)	62%	57%	61%	75%	0.347
Stellate ganglion score (range)	0.58 (0–4)	0.50 (0–3)	0.64 (0–4)	0.55 (0–3)	0.923
Cardiac denervation score, mean ± SEM (range)	0.51 ± 0.12 (0–2.8)	0.40 ± 0.22 (0–2.8)	0.52 ± 0.16 (0–2.8)	0.65 ± 0.34 (0–2.8)	0.790

*Note*: Values represent mean ± standard error of the mean (SEM) or the % of cardiac features in the number of subjects. Cardiac sympathetic denervation score was calculated using the equation *Y* = –0.4331*X + 3.244 (Figure 3G). For the details of other variables see Table 1 notes.

### 2.7 Cardiac sympathetic innervation: TOH and stellate ganglion αSYN immunoreactivities

Autonomic innervation of the heart is controlled by an extensive network of sympathetic and parasympathetic nerves (Figure S1 in supporting information). Sections containing the stellate ganglion were immunostained for αSYN and evaluated in a semiquantitative manner. The degree of immunoreactivity was scored on a scale of increasing severity from 0 to 4, with a score of 0 indicating no pathology and a score of 4 signifying severe pathology (Figure S2 in supporting information). *Post mortem* evaluation of αSYN pathology within the nervous system was used to demonstrate microscopic loss of cardiac nerves.^32^ We used the two markers to reveal if there is a causal link between αSYN immunoreactivity and cardiac denervation, and to find evidence of whether αSYN accumulation precedes any cardiac denervation.^33^, ^34^

Postganglionic sympathetic nerves (noradrenaline) are more frequent throughout the heart and relatively straightforward to evaluate while the parasympathetic cholinergic nerves are less frequent. Studies on cardiac denervation have frequently used [123I] metaiodobenzylguanidine (^123^I‐MIBG, an analogue of noradrenaline) scintigraphy to assess cardiac sympathetic denervation.^35^ Immunohistochemically, sympathetic nerves were identified by TOH immunoreactivity. Thus, sections of the myocardium from the left ventricle wall with an arteriole (50 µm in diameter) and surrounding epicardial adipose tissue in the trans‐sectional plane were evaluated for pathology (Figure S3 in supporting information). Nerves cut obliquely or on the tangential plane were generally excluded. Individual nerves and thus axon density meeting these evaluation criteria were assessed for TOH immunoreactivity. In the rare cases in which there was either no adipose tissue or no appropriate nerves present in the adipose tissue the myocardium was screened. A semiquantitative scoring system was used to assess cardiac sympathetic nerve densities. A score of 1 indicates 0% to 25%, a score of 2 indicates 26% to 50%, a score of 3 indicates 51% to 75%, and a score of 4 indicates 76% to 100% coverage of TOH reactivity within the nerve (Figure S3). The sum of TOH scores from all scanned images for each case was divided by the number of the scored nerves to calculate a mean score. Those sections with no suitable or observable nerves were omitted.

To ensure the reliability of the scoring method an intra‐rater analysis was performed by two raters. Upon screening all images, a set of 30 unique random integers was generated corresponding to 30 images in the initial screening process to be used in this statistical analysis. Agreement between the two scorers was determined by the value of Krippendorf alpha at 0.7 signifying acceptable intra‐rater agreement.

### 2.8 Statistical analysis

Raw data were analyzed using GraphPad Prism 10 software and IBM SPSS Statistics version 29.0.1.1. Standard descriptive statistics were used to describe the CogFAST samples. Unless otherwise stated, data are presented as mean ± SEM. The Shapiro–Wilk test was used for normality testing. Bivariate relationships of key characteristics — age, brain weight, degrees of stenosis, sclerosis, vascular pathology scores, number of vascular lesions, stellate ganglion pathology, and TOH scores — were examined using correlation analyses (Pearson *r*), Student *t* tests, multiple Fisher exact tests, or analysis of variance (ANOVA) as appropriate. Post hoc authentication for multiple comparisons included Tukey and Bonferroni tests. The percentages of stenosis and sclerotic index were compared between respective stroke and non‐stroke cases. The differences between variables to baseline characteristics were also compared using a Pearson chi‐squared test. *Z* scores were established using the formula: *z* = (*x* – *u*)/σ, where: *X* = raw scale score, *U* = mean, and σ = standard deviation. In all analyses, a value of *P *< 0.05 was considered statistically significant.

### 2.9 Data availability

The data supporting findings in this study are available on request from the corresponding author. The data are not publicly available due to case privacy and ethical restrictions.

## 3 RESULTS

### 3.1 CorA disease and cardiac pathology in stroke

In this study, we evaluated 75 stroke cases out of 100 records for those we had as complete clinical and pathological information as possible, with *post mortem* samples of coronary arteries and cardiac tissue (Table 1). We related various clinical and pathological features to the degree of CorA disease classified as mild, moderate, and severe. There were no differences in the mean degrees of stenosis between the arteries representing the main arterial systems of the heart, vis a vis the LD, and the main LA and RA coronary arteries (Figure 1A). The mean (± SEM) % area stenosis in the three arteries: LD, left coronary (LC), and right coronary (RC) were: 76.4 ± 1.9, 76.9 ± 1.8, and 79.2 ± 1.7 respectively (ANOVA *P *= 0.514). We noted 7% of the cases had 100% stenosis that was almost equally distributed across the three arteries. In a subsequent analysis, we averaged the % area stenosis value from all three arteries and used this as a single measure per case. The mean % stenosis in the CorAs of the whole sample was calculated to be 75.5% with a mean (± SEM) age of 87 (± 1) ranging from 65 to 99 years (Table 1). The mean % area stenosis within the mild, moderate, and severe groups was 65%, 77%, and 91%, respectively (Table 1). These values indicated that nearly 100% of the cohort had clinically defined occult CorA disease or > 50% stenosis as determined by maximal luminal diameter.

There were no differences in age, % male, or brain weights among the mild, moderate, and severe stenosed CorA disease groups (Table 1). Subjects who developed dementia post‐stroke ranged from 46% to 61% within the mild, moderate, and severe groups (*P *> 0.05). There was no correlation with degree of CorA disease and dementia or differences in either the final mean MMSE or CAMCOG scores between the groups (Table 1). In terms of VRFs, in the total group we noted 57% had a history of hypertension, 7% diabetes, and 13% hypercholesterolemia. The presence of VRFs was not related to the degree of CorA disease but it was apparent that 27% of the group exhibited more than three VRFs (Table 1).

Histopathological examination of segments of the LD, LC, and RC samples showed typical atherosclerosis to be frequently present (Figure 1).^36^ Macro‐ and microscopic examination of the CorA samples indicated five different classic types of change in the vessel walls (Figure 1B–G). More than 90% of the subjects had one type of lesion in these age groups. The frequency in the order of the CorA disease subtypes in the total sample were intimal thickening > fibrocalcific > fibrous cap (thick) > fibrous cap (thin) > other groups. However, there were substantial proportions of fibrocalcific and fibrous cap (thick) lesions. Ruptured atheromatous lesions were rare with a frequency of < 5% in the total sample.

To identify specific changes within the intima and the atheroma in the vessel walls, we immunostained segments of CorAs with CD68 as an inflammatory cell marker and isoDGR to detect degenerative protein modifications (Figure 1H–M). We found extensive accumulation of isoDGR‐modified (deamidated) proteins^12^, ^37^ in the vessel walls overlapping with CD68‐positive macrophages. This pattern is consistent with ongoing vascular protein injury and a sustained inflammatory response within the CorA atheromas, similar to that observed in carotid artery lesions.^27^

With the hypothesis that CorA and cardiac pathologies influence brain pathology, we first compared various vascular and neurodegenerative pathological indices in relation to the CorA stenosis. We found a lack of differences in scores of various cerebral neurodegenerative pathologies within the mild, moderate, and severe CorA disease stenosis groups (Table 1). Likewise, the degrees of cerebral SVD pathology, WM changes, and intracranial arterial disease were represented almost equally in the CorA disease stenosis groups (Table 1).

### 3.2 Relationship among CorA, carotid artery diseases, and SVD pathology

We next determined whether CorA stenosis was related to internal carotid artery (ICA) stenosis and whether the stenosis in both coronary and carotid artery systems was associated with brain SVD pathology and the burden of infarcts (Figure 2). Pearson analysis showed that CorA stenosis was positively correlated with ICA stenosis (*r* = 0.275, *P *= 0.032; Figure 2A) indicating a relationship between CorA disease and carotid artery disease. To test whether the degrees of patency of the CorAs and carotid arteries influenced the burden of cerebral vascular lesions, we constructed a matrix whereby the number of cerebral infarcts were calculated when the CorA and carotid arteries both exhibited mild stenosis progressing up to severe stenosis with nine possible combinations (Figure 2B and C). The violin plots and accompanying heat map illustrated that the highest number of cerebral infarcts (*n *= 91) was present in cases (*n *= 23) when both arterial systems were moderately stenosed. In this scenario, most of the infarcts were cortical (55%) and the rest were distributed in the following regional order: basal ganglia/thalamus (21%) > cerebellum/brainstem (17%) > WM (8%).

**FIGURE 2:**
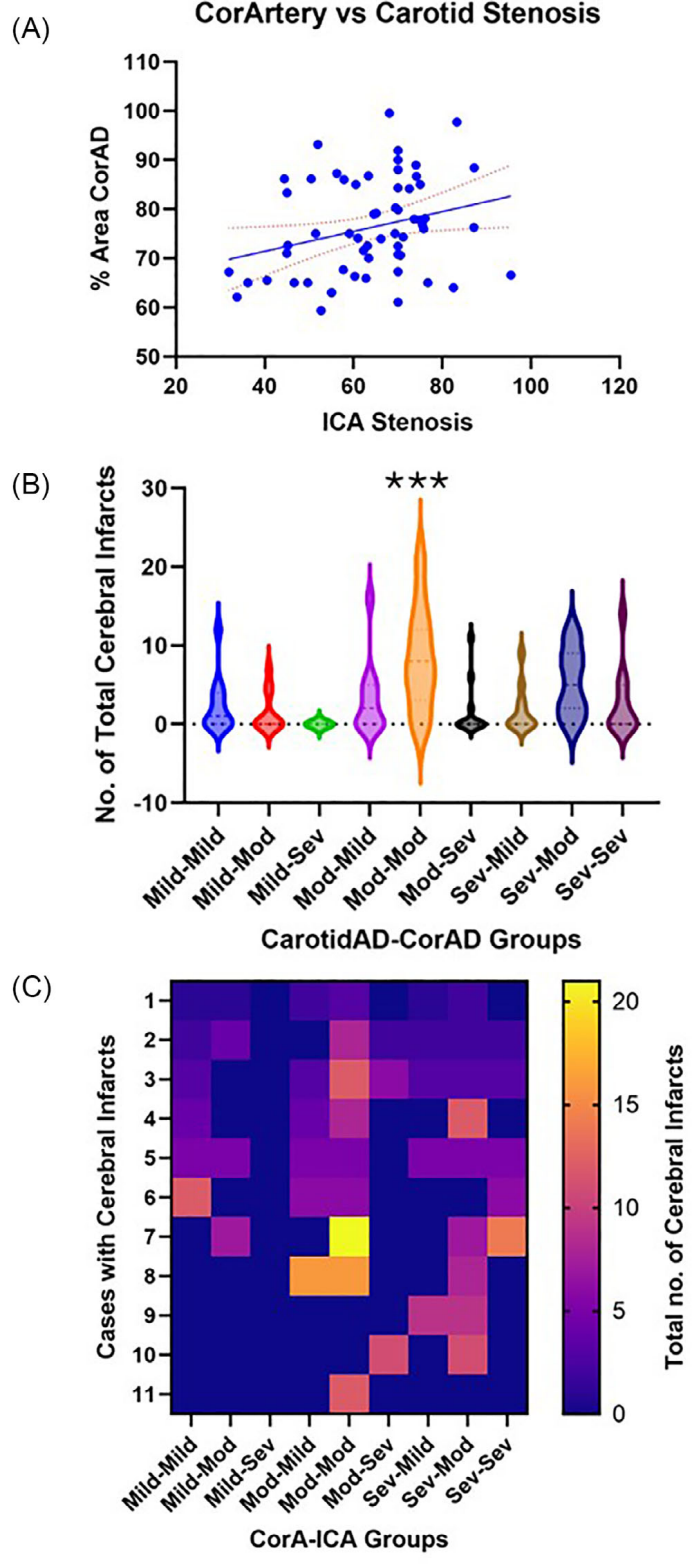
Relationship between % area coronary artery and carotid artery stenoses with features of cerebral SVD. A, Correlation showing a positive relationship between mean values of % area coronary artery and carotid artery stenoses (*r* = 0.275, *F* 4.26, dfn 1, dfd 39, *P *= 0.032). B, Violin plots to show impact of degrees of coronary artery and ICA stenosis in nine different combinations. ANOVA of VLs versus ICA stenosis groups showed significant differences *F* 5.316, df 8, *P *< 0.001. Multiple comparisons of individual groups showed differences between the following groups (CorA‐ICA): Mild–Mild versus Mod–Mod (*P *= 0.0031),  Mild–Mod versus Mod–Mod (*P *= 0.0012),  Mild–Sev versus Mod–Mod (*P *< 0.001), Mild–Sev versus Sev–Mod (*P *= 0.04),  Mod–Mild versus Mod–Mod (*P *= 0.0181), Mod–Mod versus Mod–Sev (*P *= 0.0004), Mod–Mod versus Sev–Mild (*P *= 0.0012), and Mod–Mod versus Sev–Sev (*P *= 0.0031). *** The highest number of cerebral VLs (< 5 mm infarcts) were evident when both coronary and ICAs exhibited moderate stenosis. C, Heat map to show moderate patency of the CorA and ICA were associated with a high number of cerebral infarcts in CogFAST subjects. ANOVA, analysis of variance; CogFAST, Cognitive Function After Stroke; CorA, coronary artery; ICA, internal carotid artery; Mod, moderate; Sev, severe; SVD, small vessel disease; VLs, vascular lesions.

In further analyses, we assessed the location and size of the lesions in the three groups (Figures S4 and S5 in supporting information). These lesions were characterized as class II predominantly fitting SVD per the original Newcastle classification system.^38^ The highest numbers of infarcts in these cases in which CorA disease was assessed were in the cortex with 37% in mild and 51% in moderate CorA disease cases (Figure S4A–D). In terms of the size, it was striking that small lesions < 5 mm were most prevalent throughout the brain regions ranging from 72% to 77% (Figure S5A, F, and L in supporting information). The basal ganglia and thalamus exhibited the highest numbers (up to 92%) of these small infarcts (Figure S5C, I, and N). A common pattern of the regional distribution of these small infarcts was BG (basal ganglia and thalamus) ≥ other (cerebellum and brainstem) > WM > cortex. There were no overall differences in the distribution of infarct size among the mild, moderate, and severe stenosis groups (Figure S4).

### 3.3 Cardiac pathology and CorA in CogFAST

We evaluated the frequencies of several clinical features of cardiac disease in this CogFAST cohort (Table 2). We noted that 60% of the cohort had at least one cardiac condition or trait with 47% having IHD. There were no significant trends in the degree of CorA stenosis and cardiac clinical features although there were high frequencies of heart conditions in those with moderate CorA stenosis (Table 2).

Cardiac histopathology revealed a spectrum of pathologies in the cohort. This included changes consistent with both acute and remote ischemic events comprising micro, small, and large infarcts, mild diffuse and multifocal scarring as well as severe myocardial amyloidosis (Figure 3). In many cases, we noted severe fibrosis (collagenosis), disarray (wavy fibers) and fibers with damaged or “Ninja” nuclei or stars (Figure 4A–F and legend). Quantitative analysis showed that at least 80% of the whole cohort had evidence of characteristic pathological change with 62% exhibiting myocardial infarcts (Table 2). Increasing CorA stenosis tended to be aligned with greater cardiac pathology. This was evident when cardiac pathology was scored on a semi‐quantitative scale from 0 to 3 (Table 3). We also found evidence for a positive relationship between CorA stenosis and heart weights as well as between cardiac pathology scores and myocardial infarcts (Table 3). Furthermore, the degree of cardiac pathology was strongly related to heart weight (Table 3).

**FIGURE 3:**
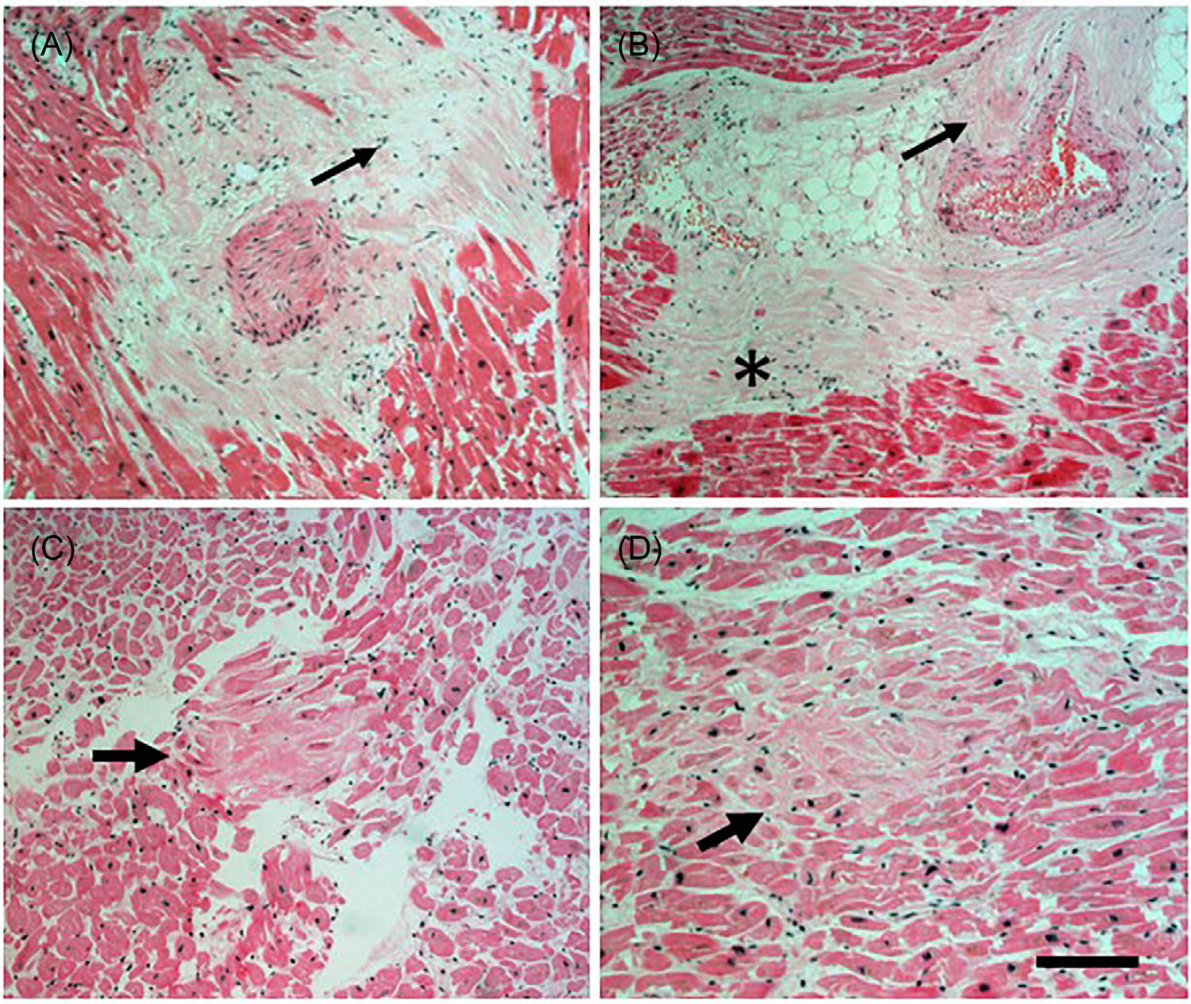
Features of chronic myocardial pathology in the Cognitive Function After Stroke cohort. A–D, Representative images showing (A), microinfarct (arrow) and peri‐infarct rarefaction; (B), post‐infarction scarring with intact arteriole (arrow) and fibrous muscle structures (asterisk) or fat infiltration; and (C–D), amyloidosis (thick arrows) with amorphous pink or eosinophilic material around muscle cells. Scale bar represents 200 µm (A–D).

**FIGURE 4:**
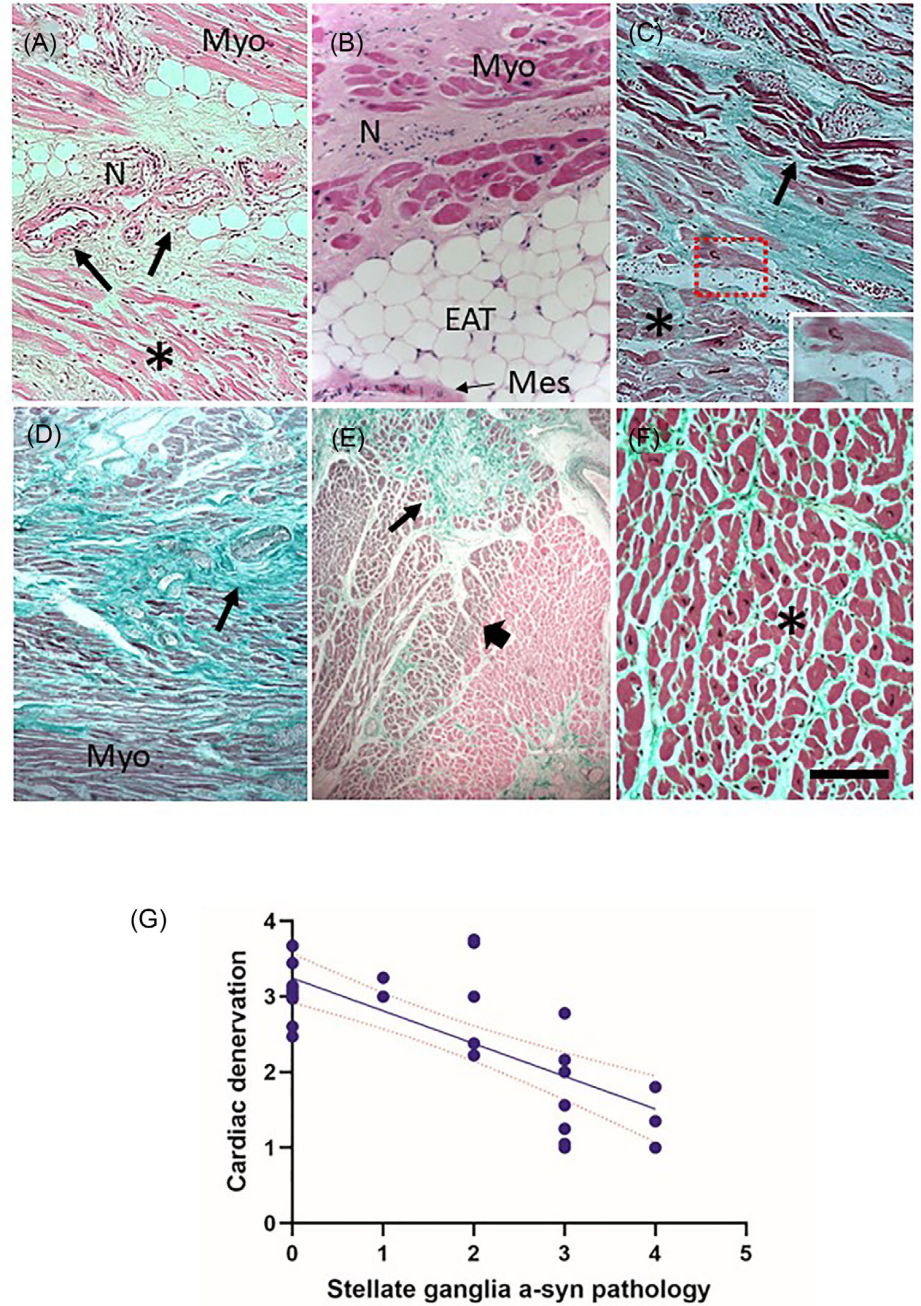
Cardiac cellular pathology in the CogFAST cohort. A–F, Representative images show different types of cardiac muscle changes caused by recent and remote infarctions. A, B, Some loss of normal fibers (asterisk) and presence of adipose tissue (EAT). Nuclei here appear almost intact. Moderate hyalinosis can also be discerned (arrows in [A]). C, Disarray shown by damaged darkly stained wavy fibers (elongated and narrow) compared to adjacent normal fibers (asterisk) and several “ninja” nuclei or stars. These nuclei assume bizarre hyperchromatic haphazard or star shapes different from the regular circular or oval normal shapes.^23^ D, E, Perivascular regions with severe fibrosis or collagenosis (arrow) between muscle fibers. E and F, Myocardial infarcted or hypoperfused region (thick arrowhead) and disrupted or necrotic tissue (asterisk) with several “ninja” nuclei. A, B, Moderate neutrophil or polymorphonuclear leucocytic infiltration (N). H&E and Masson's Trichrome stains were implemented, with Masson's Long Collagen (COL) stained in green. G, Relationship between stellate ganglion pathological changes (α‐synuclein deposition) and cardiac sympathetic innervation (TOH immunoreactivity). Scale bar represents 200 µm (A–F).CogFAST, Cognitive Function After Stroke; EAT, epicardial adipose tissue; H&E, hematoxylin and eosin; Mes, mesocardium; Myo, myocardium; N, neutrophil; TOH, tyrosine hydroxylase.

**TABLE 3:** Relationship between indices of coronary artery disease and cardiac pathology.

X variable	Y variable	Pearson *r*	*N*	95% CI for mean		*P* value^*^
				Lower bound	Upper bound	
CorA disease stenosis	Heart weight (g)	0.244	56	−0.020	0.476	0.070
	**Cardiac pathology score**	**0.412**	**75**	**0.207**	**0.584**	**0.002**
	Stellate ganglia score†	−0.010	59	−0.262	0.251	0.964
Cardiac pathology score	**Heart weight (g)**	**0.444**	**57**	**0.207**	**0.631**	**0.0005**
	**Myocardial infarct score**	**0.463**	**70**	**0.252**	**0.646**	**0.001**
	**Stellate ganglia score** ^a^	**0.267**	**71**	**0.036**	**0.471**	**0.0245**

*Note*: Results of Pearson (*r*) correlation analyses between the degree of coronary artery stenosis and heart pathology within the CogFAST cohort.

Abbreviations: CI, confidence interval; CogFAST, Cognitive Function After Stroke; CorA disease, coronary artery disease.

^*^ Significant relationships (*P *< 0.05) are shown in bold. Variances were all assumed to be equal between groups.

^a^ Stellate ganglia pathology scores were in range 0 to 4 and inversely related to tyrosine hydroxylase immunoreactivity in cardiac tissue.

### 3.4 Cardiac innervation in stroke survivors

In further analyses, we explored the status of cardiac innervation by assessing the stellate ganglion which innervates the heart via sympathetic fibers and presence of adrenergic reactivity within the cardiac tissue. Stellate ganglion pathology assessed by αSYN immunoreactivity was inversely related to cardiac denervation assessed by TOH immunoreactivity in the same samples (Figure 4G; Figures S2 and S3). Using this index, we also examined whether there was any relationship between stellate ganglion pathology or cardiac denervation and CorA stenosis in the CogFAST cohort. While we found that stellate ganglion or denervation scores were not different between the CorA stenosis groups (Table 2), we noted cardiac pathology scores were related to greater stellate ganglion pathology and lower cardiac sympathetic innervation within the CogFAST cases (Table 3). Furthermore, only 12% of the cohort had stellate ganglion (and conversely sympathetic denervation) above the score of 3, which was defined as moderate to severe αSYN immunoreactivity (Figure S2). There was no association with the presence of dementia (see below, Table 4).

**TABLE 4:** Post‐stroke no‐dementia versus post‐stroke dementia in the CogFAST cohort.

Variable	PSND	PSD	*P* value
Number^*^	33	41	–
**Heart weight (g)** ^**^ **(range)**	**445 ± 21 (275**–**689)**	**341 ± 14 (49**–**486)**	**<0.001**
Cardiac pathology score (range)	85.1 ± 2.4 (60–98)	77.3 ± 3.4 (30–98)	0.069
CorA disease (% stenosis)	75.4 ± 1.8	75.7 ± 1.4	0.880
**Z score (cardiac pathology)** ^**^	**0.35 ± 0.16**	**−0.17 ± 0.15**	**0.024**
**Myocardial infarcts (% total)** ^a^	**44.6%**	**57.3%**	**<0.001**
Stellate ganglion score (range)^b^	0.61 ± 0.18 (0–3)	0.86 ± 0.18 (0–4)	0.362
Cardiac denervation score (range)	0.61 ± 0.16 (0–2.8)	0.51 ± 0.10 (0–2.4)	0.577
CAD (% stenosis)	61.4 ± 2.8	60.1 ± 1.9	0.696
Cerebral SVD lesions	2.7 ± 0.3	2.7 ± 0.3	0.979

*Note*: Values show mean ± standard error of the mean (SEM) unless otherwise stated.

Abbreviations: CAD, carotid artery disease; CogFAST, Cognitive Function After Stroke; CorA disease, coronary artery disease; PSD, post‐stroke dementia; PSND, post‐stroke non‐dementia; SVD, small vessel disease.

* Numbers reflect complete data sets for the variables, e.g., coronary artery stenosis in the analysis.

** Independent Student *t* test (*P *< 0.001).

^a^ Pearson chi‐squared 11.52, df 1, *P *< 0.001.

^b^ Stellate ganglia pathology scores were in range 0 to 4 and inversely related to tyrosine hydroxylase immunoreactivity in cardiac tissue.

### 3.5 Cardiac pathology and dementia

Unlike ICA disease, we observed a lack of positive correlation between indices of CorA disease or stenosis and dementia status, yet noted that heart weights were smaller in the PSD group (*P *< 0.001; Table 4). However, the composite *Z* scores incorporating cardiac pathology ratings were lower in the PSD group indicating a cardiac atrophic effect (*P *= 0.024). We also noted that there were more infarcts with an increment of 13% in the PSD compared to the PSND group (*P *< 0.001). As previously reported,^27^ there were no apparent differences in other vascular measures, namely ICA disease, or the mean number of cerebral SVD lesions (Table 4).

## 4 DISCUSSION

In this clinicopathological study, we demonstrated that components of the cardiovascular system exhibit profound pathology in elderly stroke survivors.^13^ We found that first, there is high degree of obstructive CorA disease in elderly stroke survivors presenting after the first overt event. The majority of these subjects exhibited > 70% area stenosis in the main arterial systems of the heart in both demented and non‐demented post‐stroke survivors. Our results are consistent with prior autopsy series showing high (80%) prevalence of CorA disease in patients with stroke (mean age 59–72 years),^9^ although this was evident considerably at lower frequencies (52%) in clinical studies.^5^ We also found that there were no qualitative differences between the subtypes of atheromatous lesions described previously in cardiovascular and carotid arteries,^36^ but we again reiterate that there were high frequencies of fibrocalcific lesions, disarray of both αSMA positive cells and accumulation of foam cells, collagen, and lipid clefts. We also showed that profound inflammatory cellular responses occur in tandem with degenerative protein modifications such as isoDGR, which originate in extracellular matrix proteins.^37^, ^39^, ^40^

Second, we found there was a moderate relationship between CorA disease and carotid artery stenosis. Given that the arterial wall pathological features were highly similar in the CorA and carotid arteries,^27^ it is plausible that similar hemodynamic and molecular mechanisms play a role in promoting atherosclerosis within both the coronary and carotid arterial systems.^41^ A previous *post mortem* study has suggested there could be increased incidence of intracranial arterial dolichoectasia (IADE) in SVD but we did not encounter such features in our relatively small sample. That study reported that 6% of 381 patients had IADE and that SVD was more frequent in IADE‐positive than in IADE‐negative patients (45% vs. 18%).^41^ We did not investigate the entire arterial tree from the heart to small intracranial arteries to further explore this relationship, but our observations suggest there is a degree of association. However, this is in accord with a previous autopsy study suggesting that coronary plaques and stenoses were apparent in 84% and 47%, respectively, of cases in the presence of ICA stenosis.^9^ The occurence of CorA plaques or stenosis even in the presence of severe ICA stenosis may not always predict atherosclerosis in the cerebral arteries.^27^ Irrespective, measures that reduce CorA disease and hence MI would also be important in reducing carotid artery stenosis or atheromas and maintain cerebral perfusion.

Third, our matrix analysis indicated that high numbers of cerebral infarcts, albeit mostly small cortical lesions (< 5 mm) akin to SVD, were present when there was moderate CorA and carotid artery stenosis. If the main cause of these infarcts is attributed to cardioembolic or atherothrombolic events it might be expected that cases with more severe degrees of stenosis both in the CorA and carotid arteries would result in greater numbers of infarcts. This does not take into account the pathological status of other vascular sites en route such as the aortic arch, which is a frequent location for atherosclerotic plaques.^42^, ^43^ However, these findings suggest some relationship between the compromised arterial systems of coronary and carotid arteries in promoting infarcts. Increased infarcts could also be the result of a combination of hemodynamic compromise promoted by serial stenoses as well as artery‐to‐artery embolization,^44^ as suggested by a previous clinical study in which cerebral superficial and border zone infarcts were more common in patients with concurrent extracranial and intracranial stenoses.

Different than our previous studies in carotid arteries,^27^, ^28^ we could not find a direct significant association between CorA stenosis and measures of SVD or cerebral neurodegenerative pathologies. Needless to say, this suggests that patency of the carotid arteries is critical for the perfusion of the brain and that the CorA may influence cerebral perfusion indirectly through alteration in the heart ejection fraction or cardiac output.

Fourth, total cardiac pathology described by degenerative changes and infarcts delineated post‐stroke survivors who developed dementia (PSD) from those who remained stable (PSND). Foremost, we found that heart weights or sizes were smaller in PSD subjects. It is likely heart weights were the main driver in the composite *Z* scores suggesting greater atrophy of the heart in subjects who develop delayed dementia after stroke. In the previous autopsy study, heart weights were significantly smaller in subjects with neurological diseases other than stroke with ≈ 30% that included neurodegenerative and demyelinating disorders known to be associated with dementia.^9^

Finally, MI is a risk for ischemic strokes as well as death.^45^ We note > 50% of the stroke survivors had myocardial infarcts and that there was a greater frequency of infarcts in the PSD compared to PSND subjects. Incidentally, we also detected several more myocardial infarcts at *post mortem* than those suggested by the clinical records.^9^ Correlation analysis also showed that total cardiac pathology scores were associated with not only heart weights and stellate ganglion scores but also myocardial infarcts. In addition, the burden of vascular pathology and minimal neurodegenerative changes mainly in the form of AD pathology were similar in the PSND and PSD groups.^27^ It is thus conceivable that differences in overall cardiac pathology measures between the two groups are authentic. This reasoning is compatible with reports suggesting cognitive impairment is common in older people of age > 67 years with heart failure and is associated with risk of dementia after adjustment for age, education level, net worth, and prior stroke.^46^ Similarly, in a Finnish nationwide study MI was associated with faster declines in global cognition, memory, and executive function over time.^47^ Therefore, it is not unlikely that cardiac output in terms of stroke volume, heart rate, or both affecting pulse wave velocity^48^ is compromised in stroke patients with SVD who develop dementia.^49^

We note several strengths in our study. This is clearly the largest study of its kind on the cardiovascular system and the brain with detailed analyses. We do not know of any collection in which the brain as well as prominent vessels and the heart have been examined in detail as described. We not only demonstrate that there is a high burden of cardiovascular pathology including CorA disease in older stroke survivors but those who developed dementia were prone to have even greater cardiac atrophy. We also recognize some limitations. The study would have been different with regard to the clear role of CorA stenosis and cerebral SVD or neurodegenerative pathology if we had had a larger sample. The collection of cardiovascular and brain tissues in longitudinal prospective studies, which are extremely informative, has its challenges but can be achieved with greater resources and forward planning. Although our cardiac pathology results are robust enough, perhaps as a strong preliminary observation it remains unclear whether heart insufficiency or cardiac output compromises cerebral perfusion to a sufficient extent to influence degenerative pathologies in prodromal or earlier stages of dementia. We further accept that given the multiple comparisons made during the analysis in this study, it is possible that some of the results could be a chance effect or an overestimation of the findings.

In summary, we provide pathological evidence for the high severity of CorA disease in older stroke survivors. We suggest there is a relationship, albeit moderate, between CorA disease and ICA disease. We also provide evidence to suggest that the overall cardiac pathology underscores the development of dementia in stroke survivors. Our observations implicate maintenance of typical cardiovascular health by modifying several risk factors for stroke and coronary heart disease such as physical inactivity, poor diet, smoking, excessive alcohol consumption, sleep disorders, hypertension, diabetes, obesity, and dyslipidemia, which are vital to address to prevent vascular events, maintain cerebral perfusion, and retain cognition.^50^

## AUTHOR CONTRIBUTIONS

Yoshiki Hase: analysis, interpretation, acquisition of data, editing of manuscript. Tuomo Polvikoski: case diagnosis and acquisition of data. Dan Jobson: acquisition of data, analysis, and editing of manuscript. Klarizze Premarion: acquisition of data and analysis. Ethan Tutter: acquisition of data and interpretation. Erika Kitajima: acquisition of data and analysis. Pazhanichamy Kalailingam: acquisition of data and interpretation. Xavier Gallart‐Palau: revising the manuscript and interpretation of data. Siu Kwan Sze: revising the manuscript and interpretation of data. Louise Allan: clinical data analysis and editing of the manuscript. Raj N. Kalaria: drafting, revising the manuscript, interpretation of data, diagnosing the cases, and obtaining funding.

## FUNDING INFORMATION

Our work is supported by grants from the UK Medical Research Council (MRC, G0500247), Newcastle Centre for Brain Ageing and Vitality (BBSRC, EPSRC, ESRC and MRC, LLHW), and Alzheimer's Research (ARUK). Tissue for this study was collected by the Newcastle Brain Tissue Resource, which is funded in part by a grant from the UK MRC (G0400074), by the Newcastle NIHR Biomedical Research Centre in Ageing and Age‐Related Diseases award to the Newcastle upon Tyne Hospitals NHS Foundation Trust, and by a grant from the Alzheimer's Society and ARUK as part of the Brains for Dementia Research Project. L.A. is supported by the Peninsular NIHR Applied Research Collaboration and the Exeter NIHR Biomedical Research Centre. The views expressed are those of the authors and not necessarily those of the NIHR or the Department of Health and Social Care.

## CONFLICT OF INTEREST STATEMENT

The authors have no disclosures or conflicts of interest in relation to this manuscript. Author disclosures are available in the Supporting Information.

## ETHICAL APPROVAL

Ethical approvals were granted by local research ethics committees of the Newcastle upon Tyne Foundation Hospitals Trust and were in accord with the Declaration of Helsinki. Permission for use of cardiovascular and brain tissues for *post mortem* research was also granted by consent from next of kin or family. All tissues were obtained from the Newcastle Brain Tissue Resource.

## CONSENT STATEMENT

All subjects (or next of kin) enrolled in the CogFAST study as well those designated as neurological and normal aging controls provided informed consent for both the clinical study and for brain donation.

## Supporting information




**Supporting Information**: alz71979‐sup‐0001‐ICMJE.pdf

**Supporting Information**: alz71979‐sup‐0002‐SuppMat.pdf [Please find updated Supplementary file]
