# Supplementary material for: Coronary artery disease and cardiac pathology in cerebral small vessel disease and post‐stroke dementia: Implications for Alzheimer's disease
Source: Alzheimers Dement. 2026 Nov 6;22(11):e71979. doi: 10.1002/alz.71979 (PMC13741860; doi:10.1002/alz.71979)

Supplementary Material (5 Figures)

Supplementary Figure 1: Schematic showing a simplified network of the cardiac sympathetic innervation.

Supplementary Figure 2: Immunoreactivity of α-synuclein (α-SYN) in the stellate ganglion. A-D, show typical images with increasing α-SYN immunoreactivity and the scoring system. Magnification bar: 100 μm.

Supplementary Figure 3: Immunoreactivity of tyrosine hydroxylase (TOH) in sections of α-synuclein in cardiac tissue. A-D, show typical images with increasing TOH immunoreactivity in sections of the myocardium as well as surrounding epicardial adipose tissue. Positive immunoreactivity is indicated by TOH reactivity within brown circles which was scored from 0 to 4. Magnification bar: 100 μm.

Supplementary Figure 4: Pie charts showing location of brain infarcts in subjects with mild, moderate and severe coronary artery stenosis. A, Total % of cerebral infarcts by location. B-D, Distribution of infarcts in four different brain regions including cortex, white matter (WM), basal ganglia and thalamus (BG) and other (brainstem and cerebellum) in cases with mild (B), moderate (C) and severe (D) coronary artery disease. Most infarcts were observed in the cortex, BG and thalamus, WM and other regions across moderate or severe stenosis subjects.

Supplementary Figure 5: Pie charts showing the size of brain infarcts in subjects with mild, moderate and severe CorA stenosis. A-P, Distribution of brain infarcts size in patients with mild, moderate and severe CorA stenosis. Majority of the infarcts were <5 mm regardless of the degree of CorA stenosis. Abbreviations: CA, carotid artery;

CorA, coronary artery; Mod, moderate; Sev, severe; St, stenosis; SVD, small vessel disease; VL, number of vascular lesions; VS, vascular SVD pathology scores.[17]

Supplementary Figure 1:

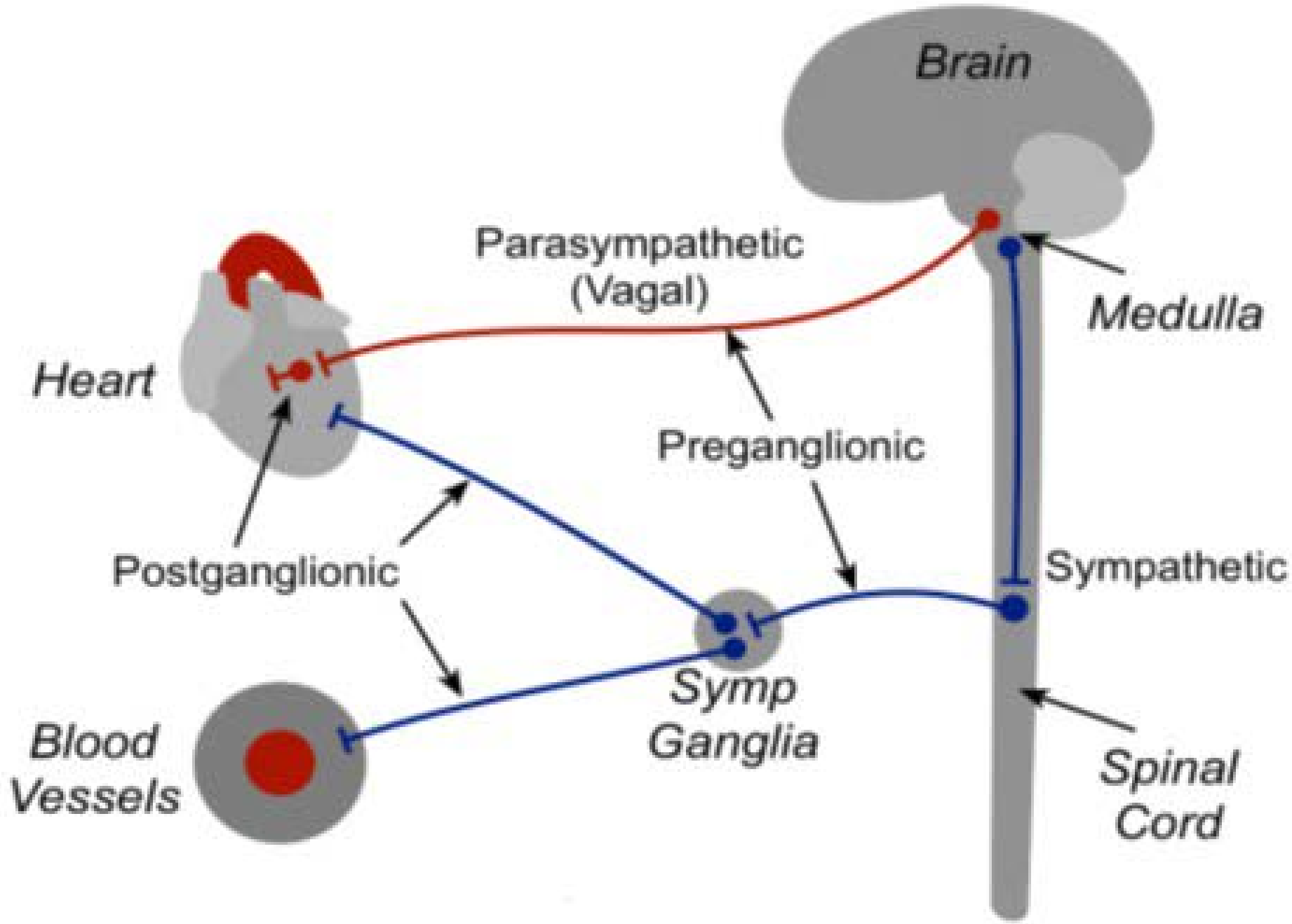

Cardiac Sympathetic Innervation: Illustration of the simplified network of the autonomic innervation of the heart

Supplementary Figures 2 and 3

2 Grading stellate ganglia pathology by α-SYN immunoreactivity

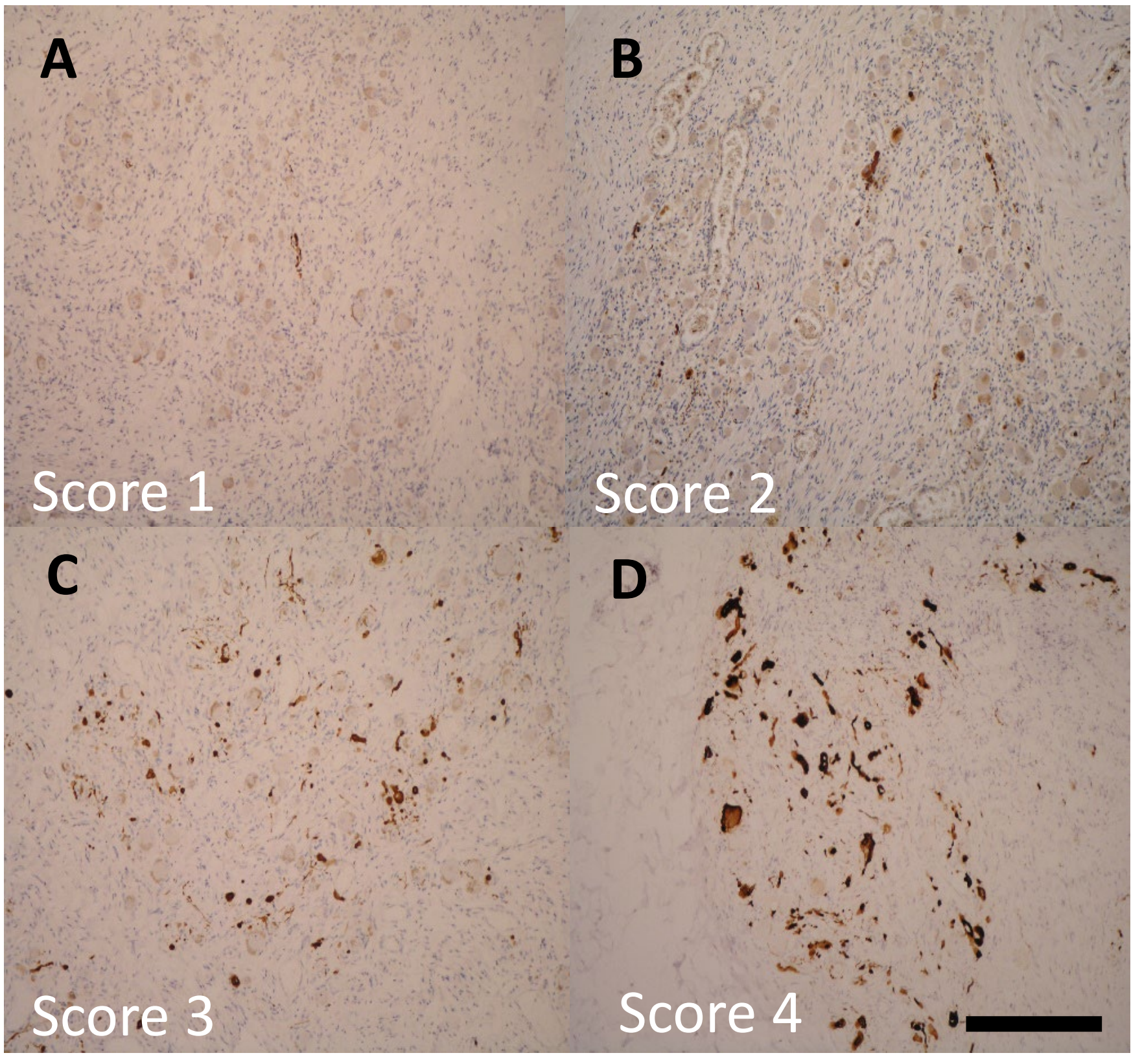

3 Grading Cardiac Innervation with pathology by Tyrosine Hydroxylase (TOH) immunoreactivity

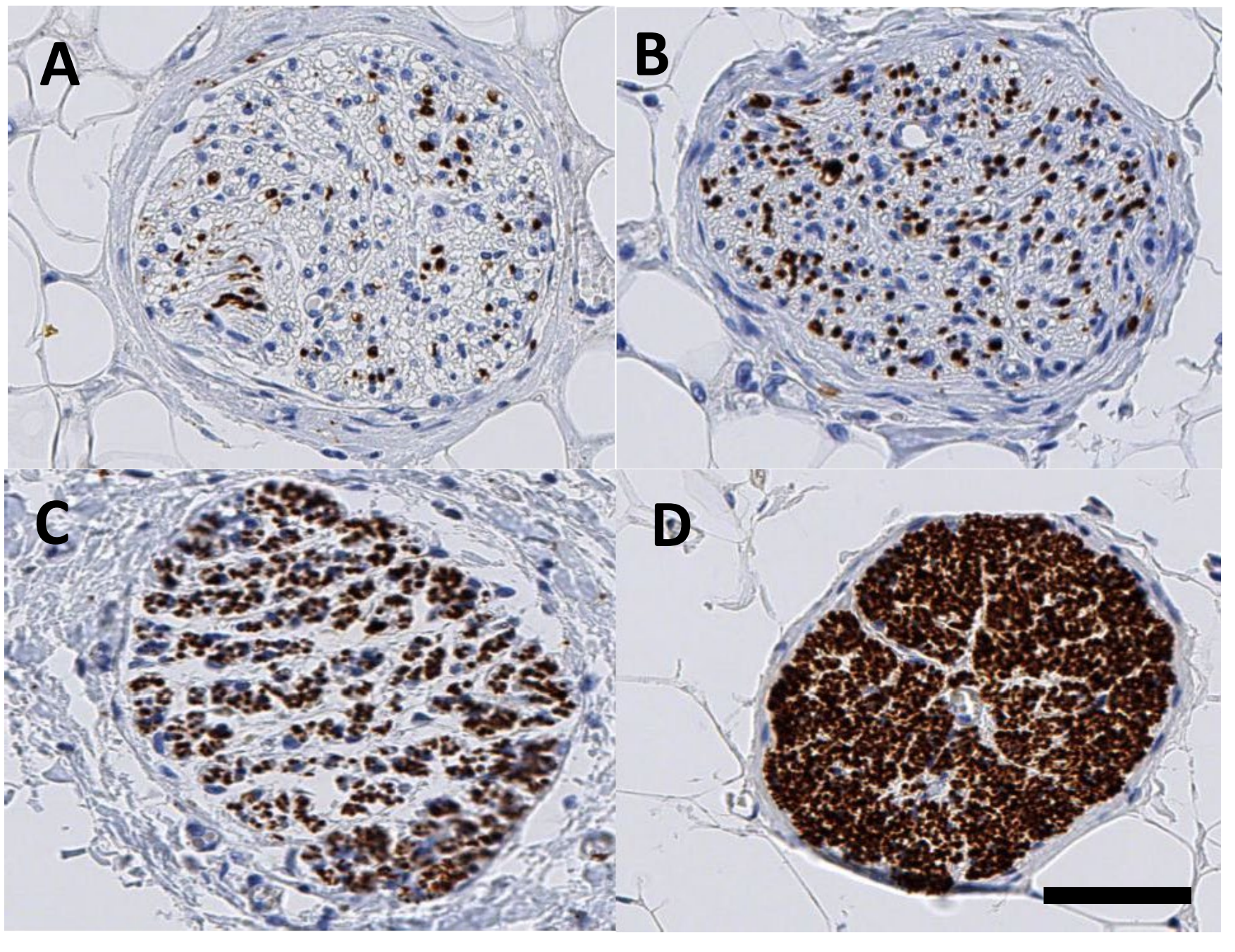

Supplementary Figure 4

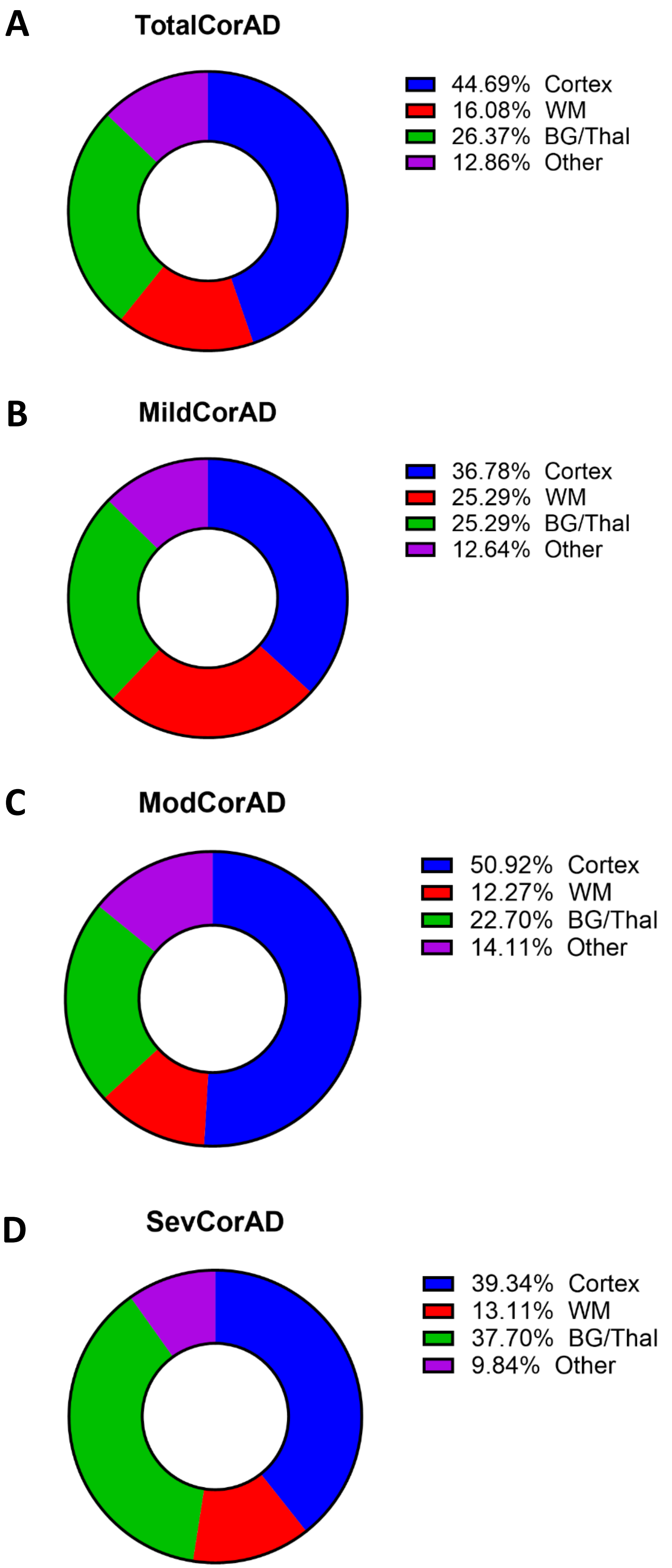

Supplementary Figure 5

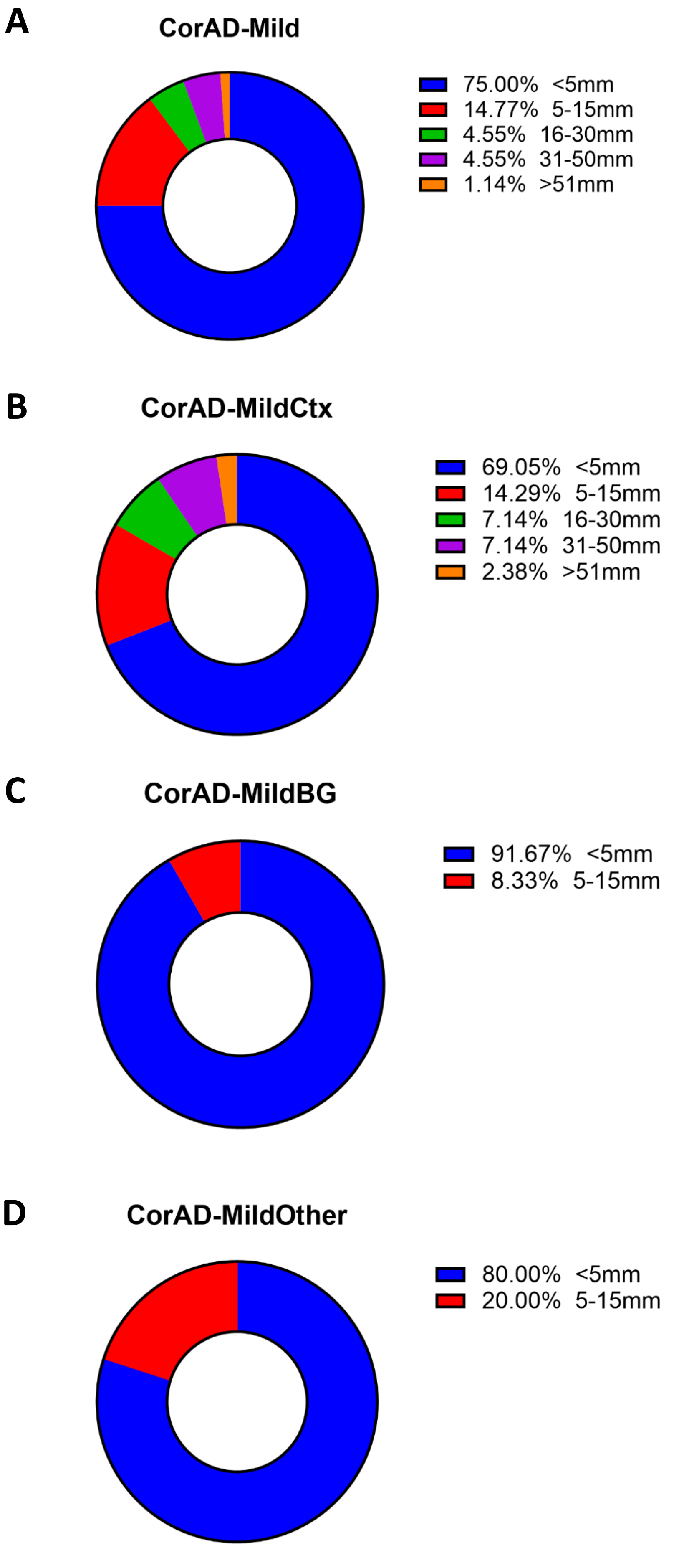

Supplementary Figure 5

E **CorAD-MildWM**

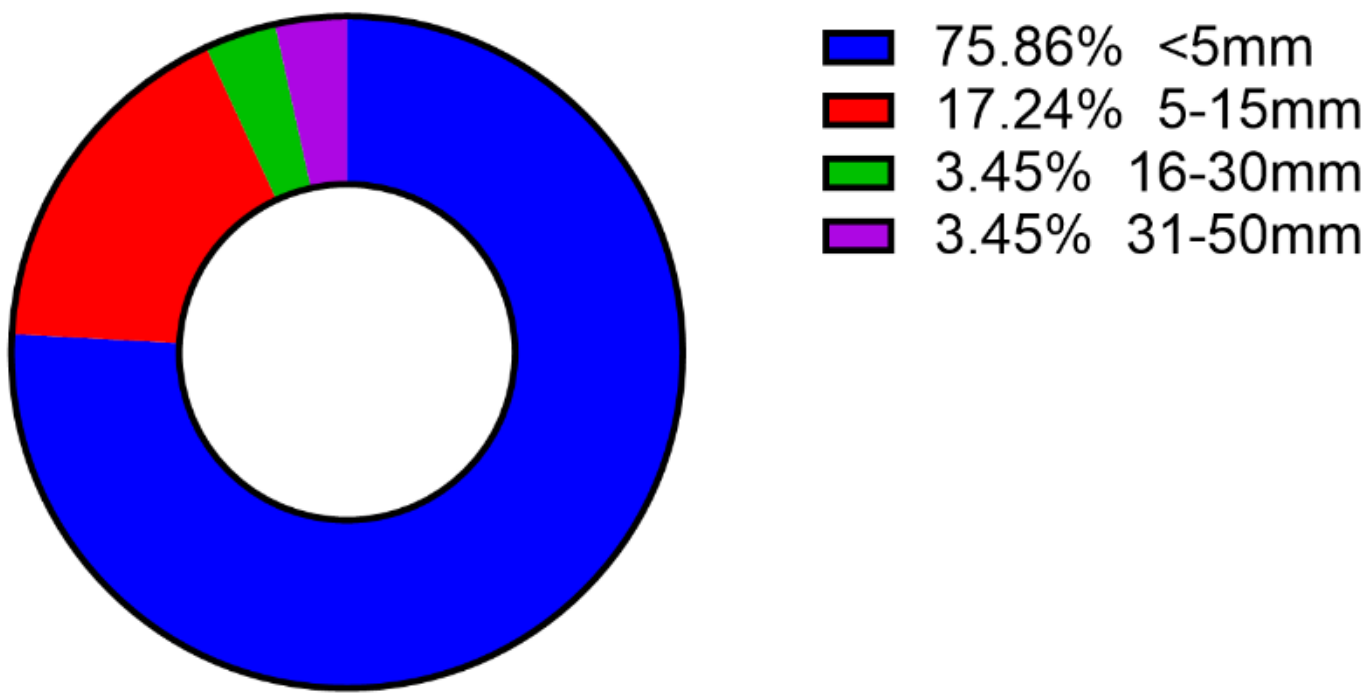

F **CorAD-Mod**

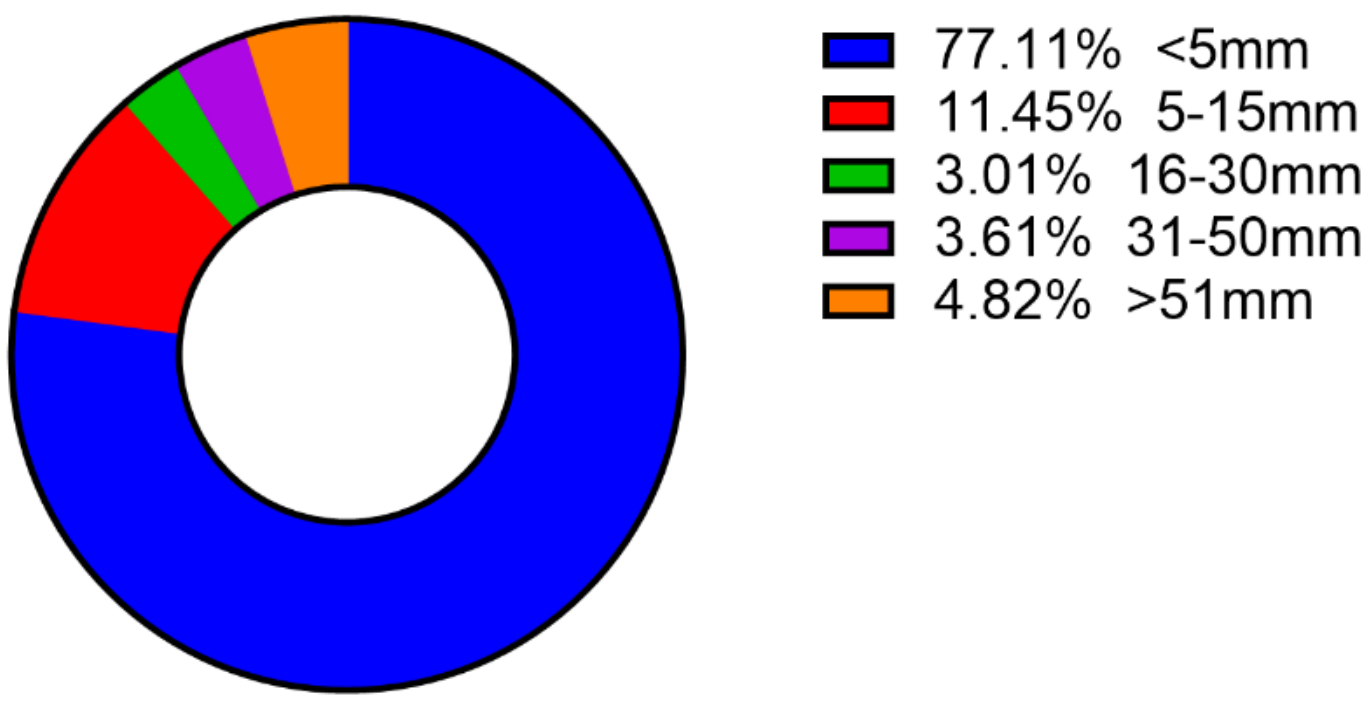

G **CorAD-ModCtx**

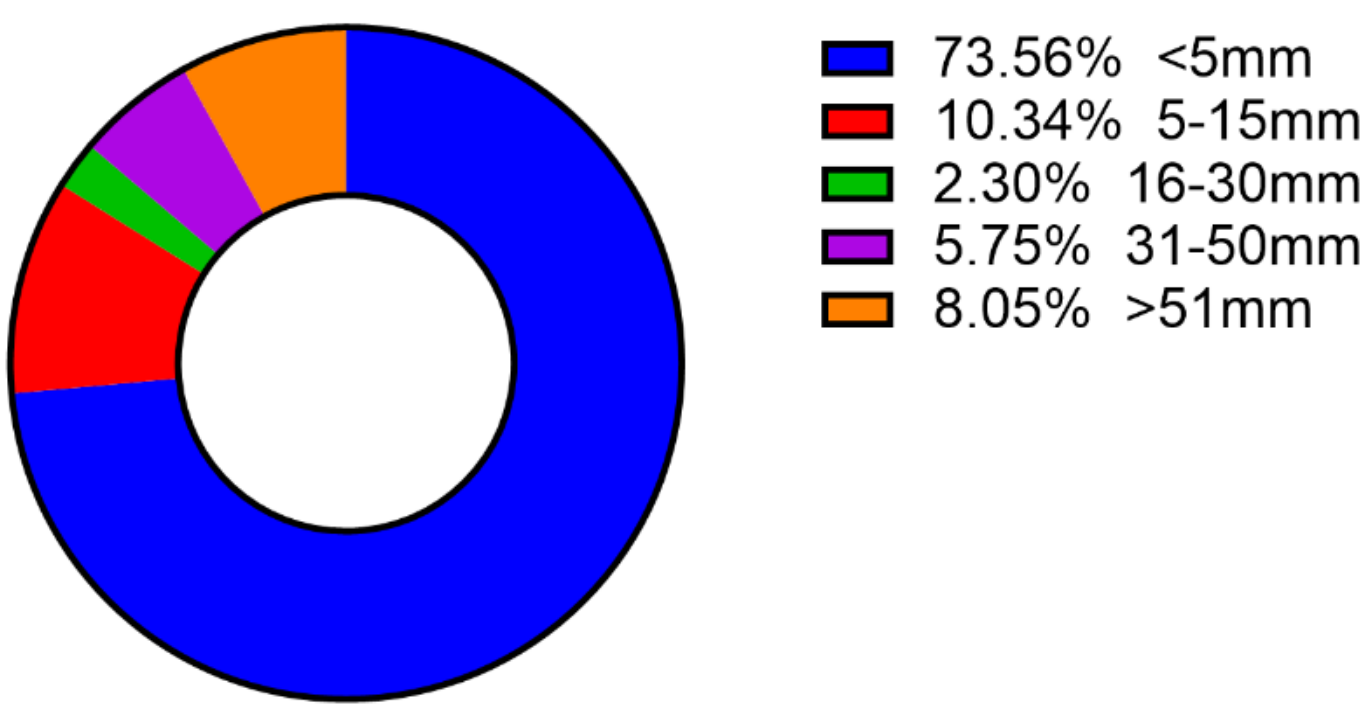

I **CorAD-ModBG**

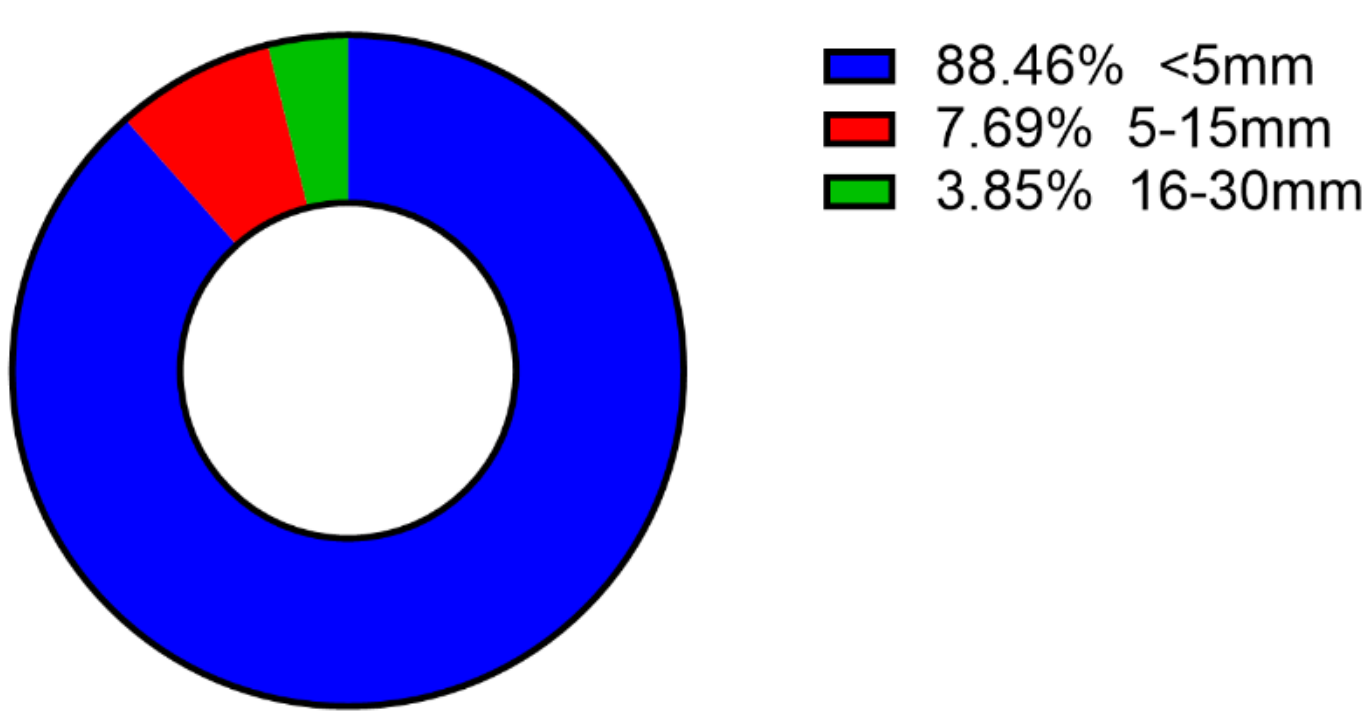

Supplementary Figure 5

J CorAD-MModOther

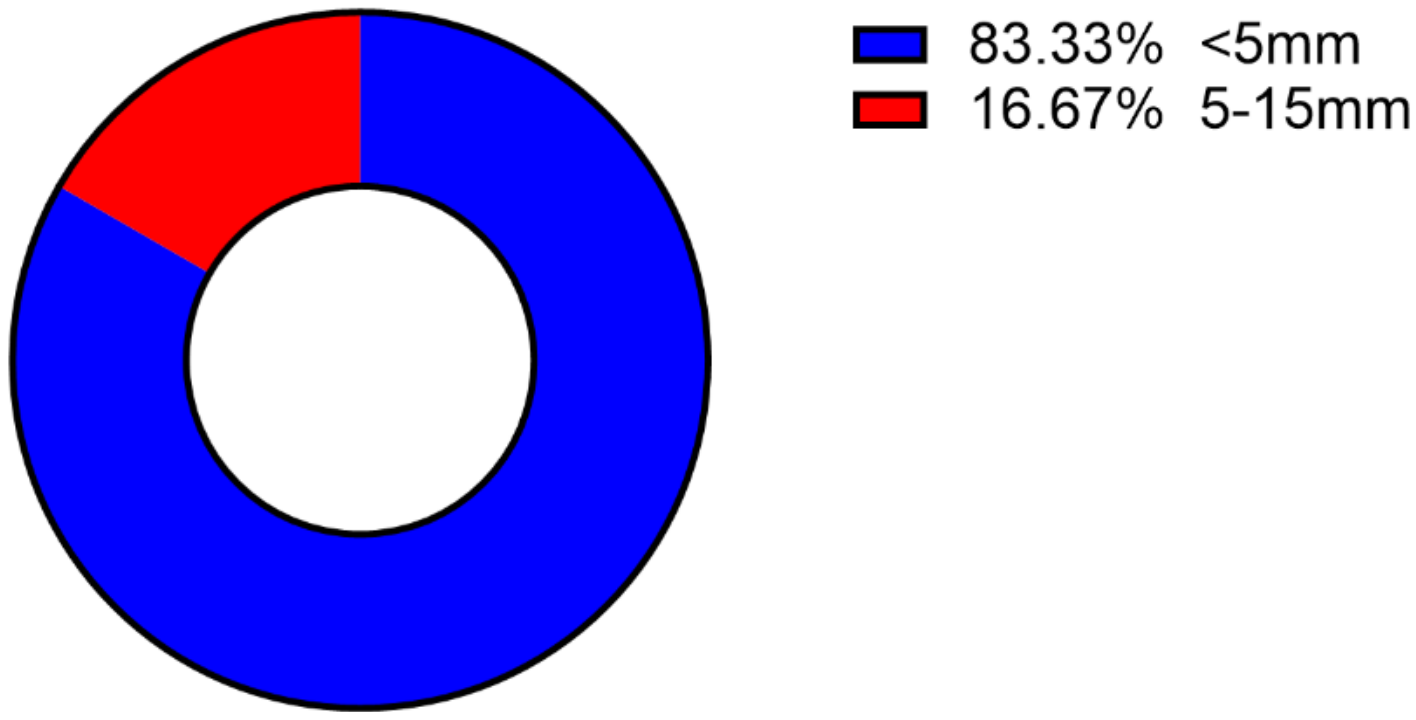

K CorAD-ModWM

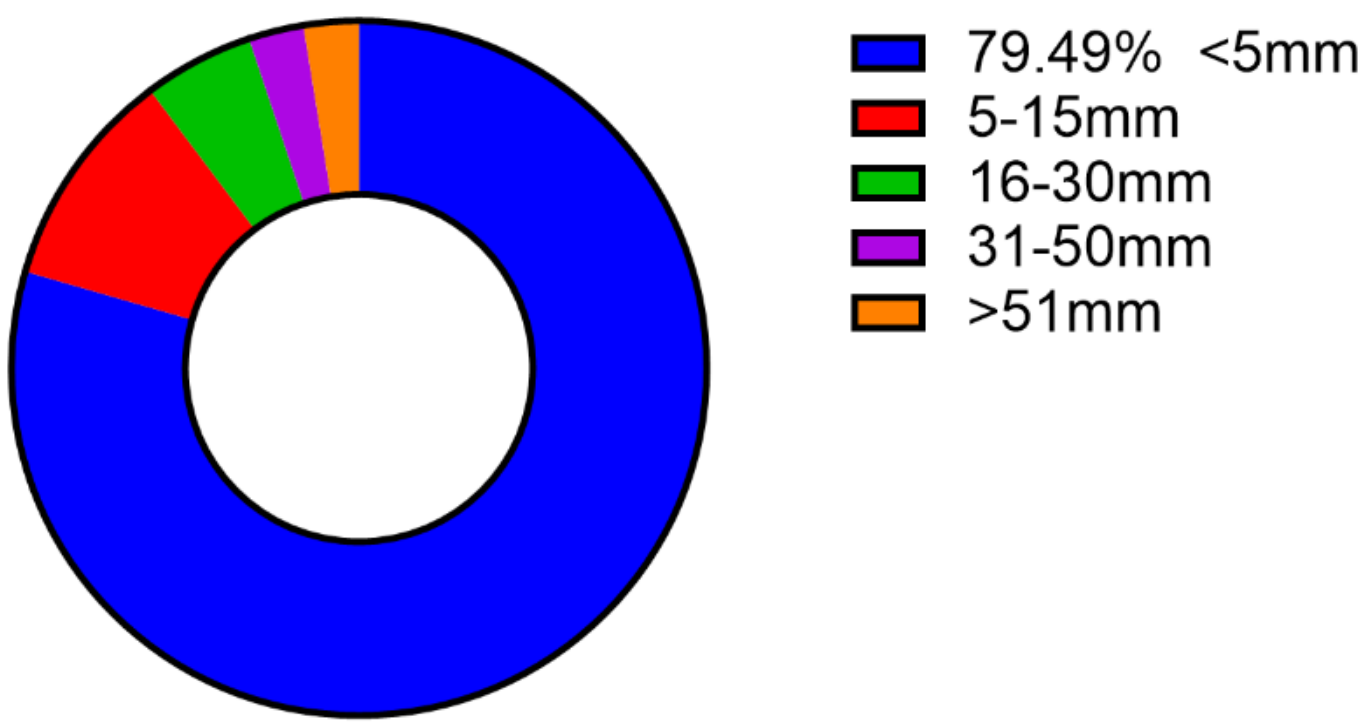

L CorAD-Sev

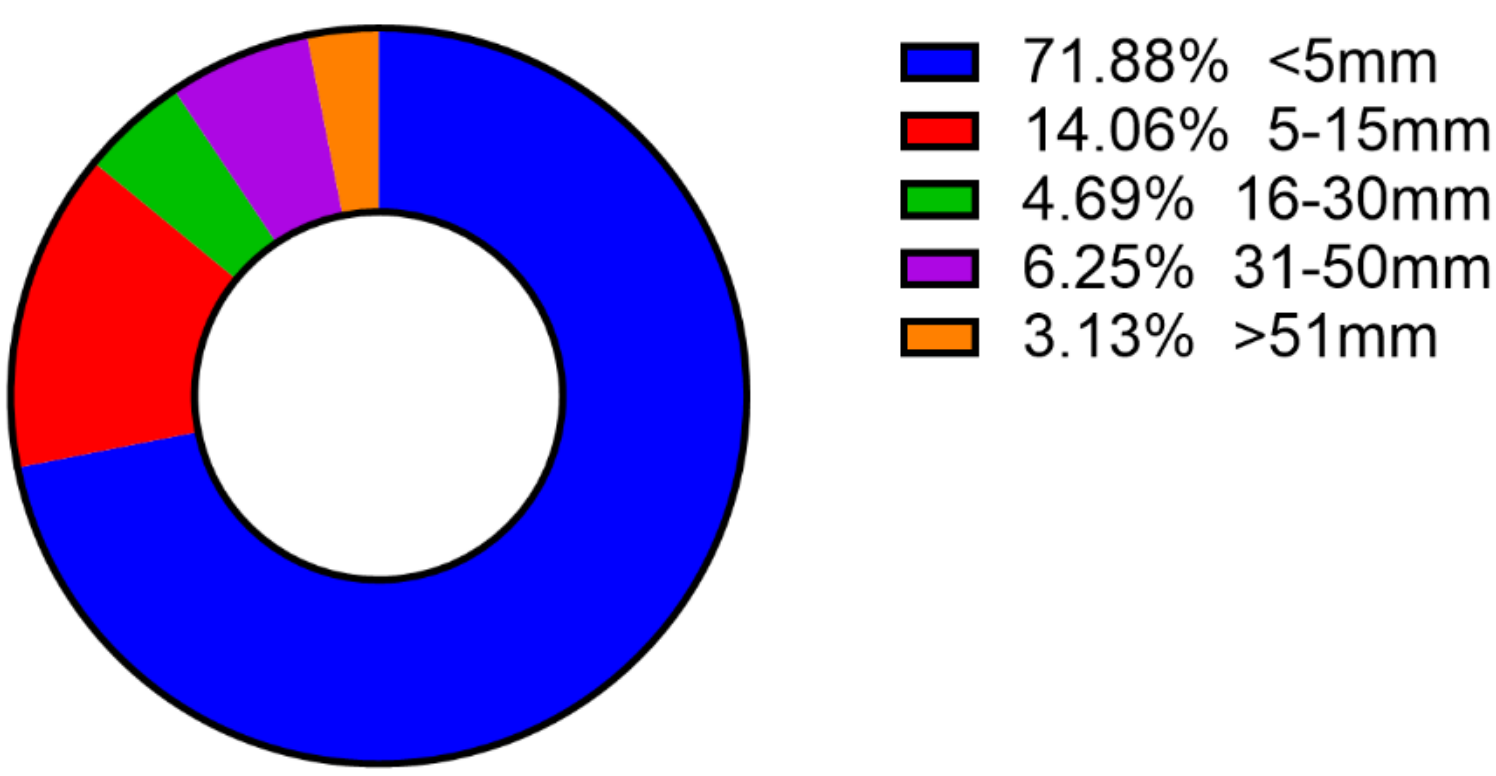

M CorAD-SevCtx

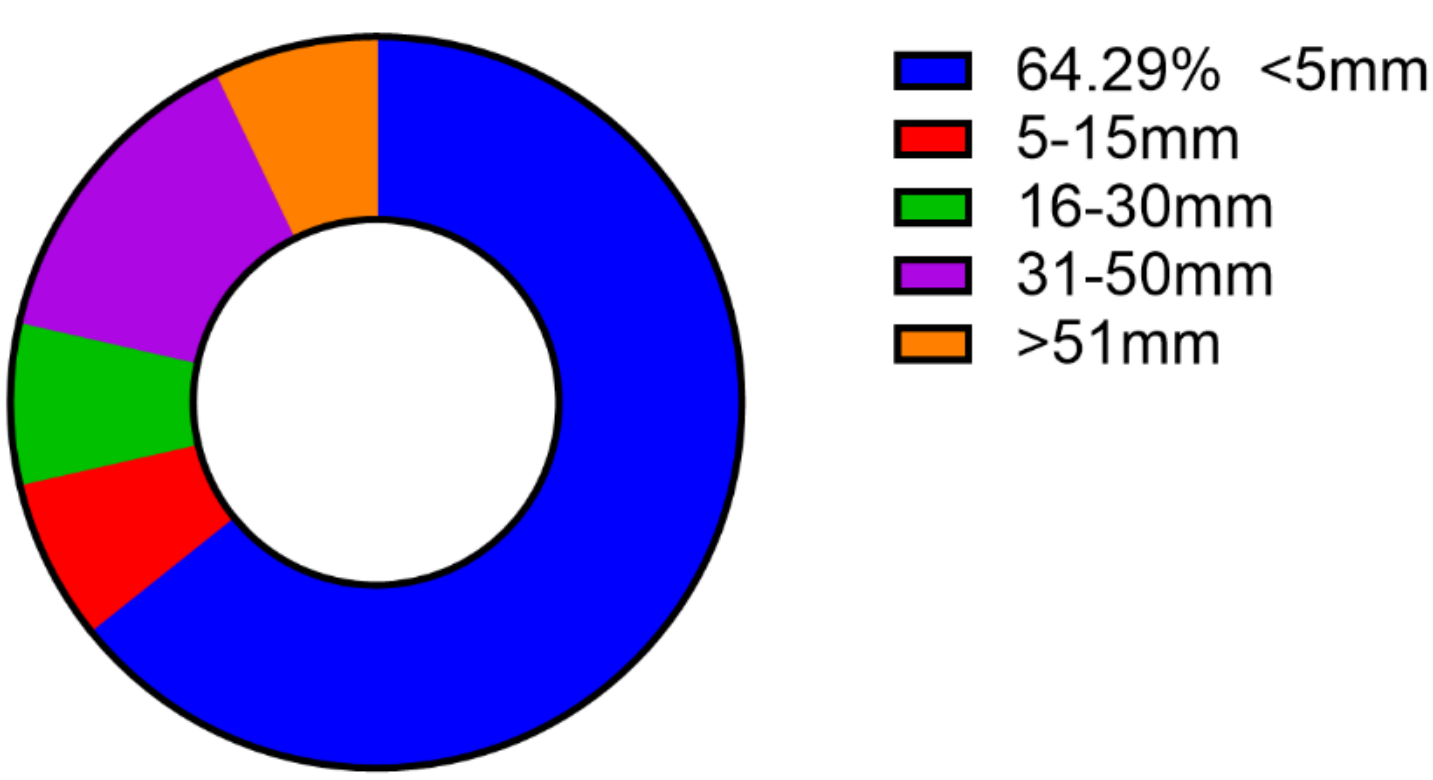

Supplementary Figure 5

N CorAD-SevBG

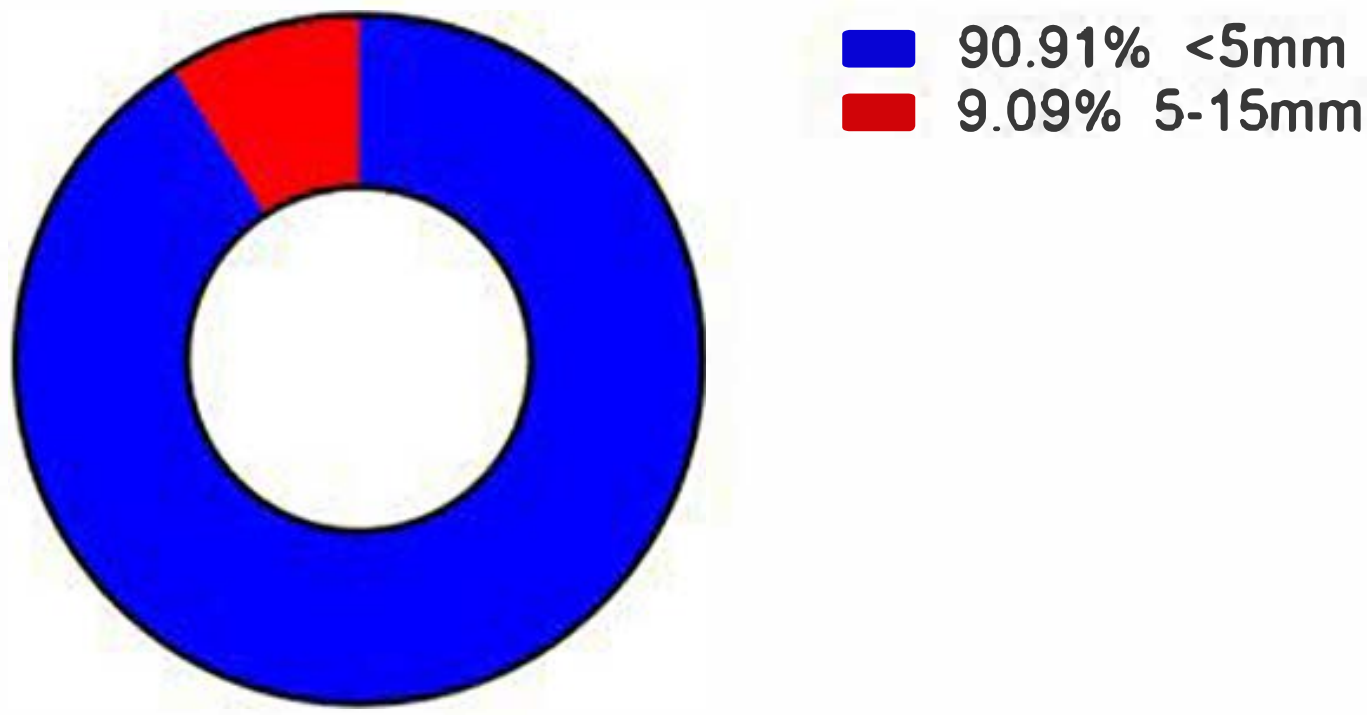

O CorAD-SevOther

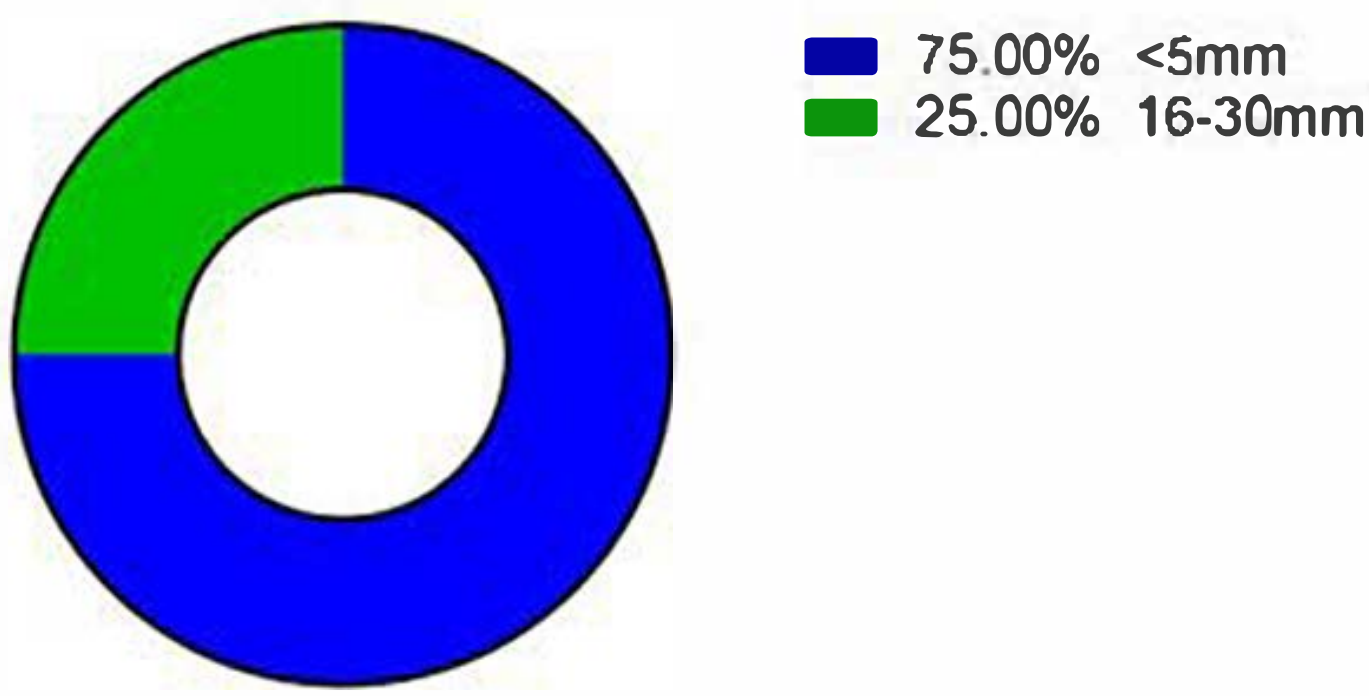

P CorAD-SevWM

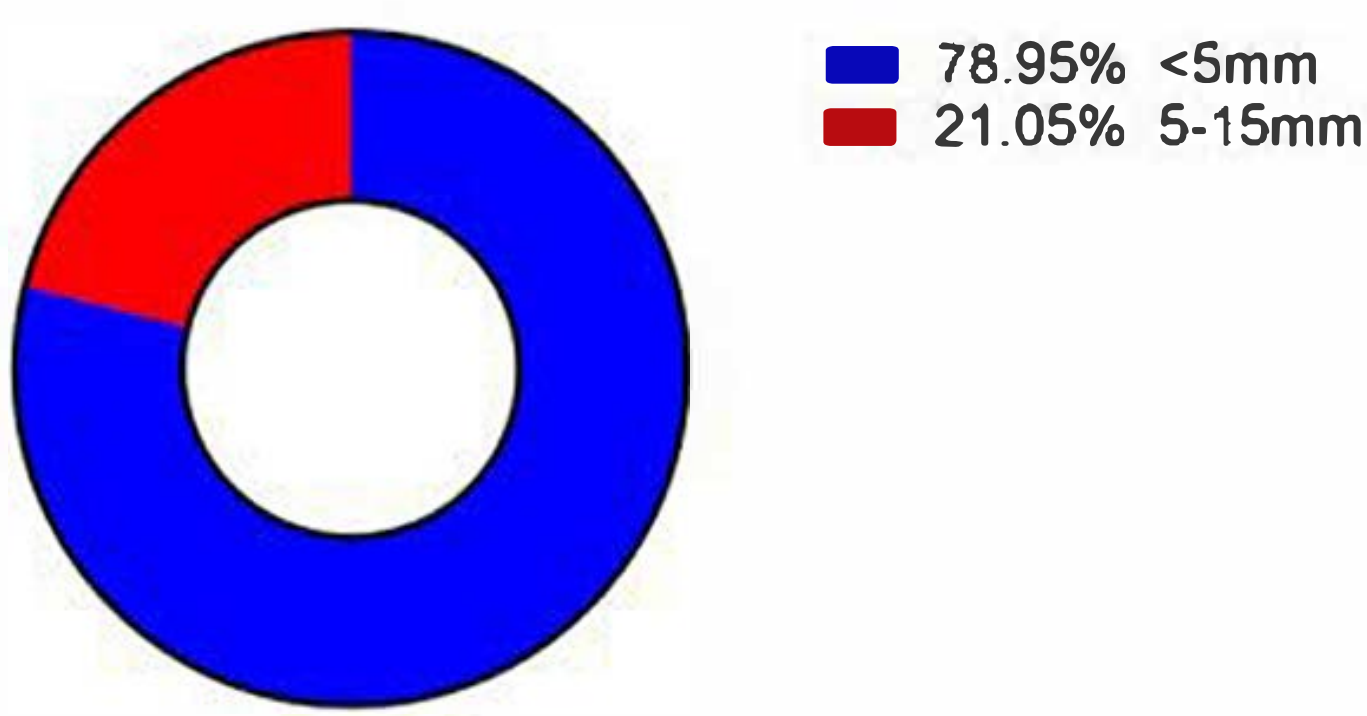

Supplement: Supplementary file 1 [file ALZ-22-e71979-s001.pdf]
